# Sensitivity, Specificity, and Public-Health Utility of Clinical Case Definitions Based on the Signs and Symptoms of Cholera in Africa

**DOI:** 10.4269/ajtmh.16-0523

**Published:** 2018-02-26

**Authors:** Johara Nadri, Delphine Sauvageot, Berthe-Marie Njanpop-Lafourcade, Cynthia S. Baltazar, Abiba Banla Kere, Godfrey Bwire, Daouda Coulibaly, Adele Kacou N’Douba, Atek Kagirita, Sakoba Keita, Lamine Koivogui, Dadja E. Landoh, Jose P. Langa, Berthe N. Miwanda, Guy Mutombo Ndongala, Elibariki R. Mwakapeje, Jacob L. Mwambeta, Martin A. Mengel, Bradford D. Gessner

**Affiliations:** 1 Agence de Medecine Préventive, Paris, France;; 2 Instituto Nacional de Saúde, Ministry of Health, Maputo, Mozambique;; 3 Institut National d’Hygiène, Lomé, Togo;; 4 Ministry of Health, Lomé, Togo;; 5 Control of Diarrheal Diseases, Community Health Department, Ministry of Health, Kampala, Uganda;; 6 Institut National d’Hygiène Publique, Abidjan, Côte d’Ivoire;; 7 Institut Pasteur de Côte d’Ivoire, Abidjan, Côte d’Ivoire;; 8 Central Public Health Laboratory, Ministry of Health, Kampala, Uganda;; 9 Division Prévention et Lutte contre la Maladie, Ministry of Health, Conakry, Guinea;; 10 Institut National de Santé Publique, Conakry, Guinea;; 11 Division de l’Epidémiologie, Ministry of Health, Lomé, Togo;; 12 Institut National de Recherche Biomédicale, Kinshasa, Democratic Republic of Congo;; 13 Division Provinciale de la Santé du Nord Kivu, Goma, Democratic Republic of Congo;; 14 Epidemiology and Diseases Control Section, Preventive Department, Ministry of Health, Community Development, Gender, Elderly and Children, Dar es Salaam, Tanzania;; 15 Curative Department, National Health Laboratory Quality Assurance and Training Center, Ministry of Health, Community Development, Gender, Elderly and Children, Dar es Salaam, Tanzania

## Abstract

During 2014, Africa reported more than half of the global suspected cholera cases. Based on the data collected from seven countries in the African Cholera Surveillance Network (Africhol), we assessed the sensitivity, specificity, and positive and negative predictive values of clinical cholera case definitions, including that recommended by the World Health Organization (WHO) using culture confirmation as the gold standard. The study was designed to assess results in real-world field situations in settings with recent cholera outbreaks or endemicity. From June 2011 to July 2015, a total of 5,084 persons with suspected cholera were tested for *Vibrio cholerae* in seven different countries of which 35.7% had culture confirmation. For all countries combined, the WHO case definition had a sensitivity = 92.7%, specificity = 8.1%, positive predictive value = 36.1%, and negative predictive value = 66.6%. Adding dehydration, vomiting, or rice water stools to the case definition could increase the specificity without a substantial decrease in sensitivity. Future studies could further refine our findings primarily by using more sensitive methods for cholera confirmation.

## INTRODUCTION

Cholera remains a major public-health issue in developing countries. In Africa, 3,221,050 suspected cases were notified to the World Health Organization (WHO) from 1970 to 2011, which represented 46% of all suspected cases reported worldwide.^1^ During 2014, Africa reported 105,287 cases, which represented an increase of 87% compared with the previous year^2^; The Democratic Republic of Congo (DRC), Ghana, and Nigeria recorded 83% of all African cases.^3^ Case fatality ratios were higher than 5% only in African countries and included Cameroon, Côte d’Ivoire, Guinea Bissau, and Kenya and Africa recorded 84% of deaths globally. The high values for disease burden in sub-Saharan Africa, however, may underestimate substantially the total disease burden. For example, some countries likely under-notified cases possibly because of fear of stigmatization: Gabon and Central African Republic did not report any cases from 2008 and 2004, respectively, even though their neighboring countries were regularly affected with cholera epidemics.^4,^^5^

From 75% to 80% of persons infected with cholera do not develop symptoms, but may still represent an important source of transmission.^6^ The primary symptoms among affected persons are acute watery diarrhea and vomiting, which may lead to severe dehydration.^7,^^8^ The mean incubation period varies from several hours to 5 days.^8,^^9^

Efficient cholera surveillance and accurate disease burden estimation rely on a clear case definition with high sensitivity and specificity. The current case definition for cholera in epidemic settings recommended by WHO was shown in Haiti to have high sensitivity (91%; 95% confidence interval [CI]: 90–93%) but low specificity (43%; 95% CI: 40–47%).^10^ Moreover, information on the performance of various case definitions is lacking in Africa and may differ from other areas due to differences in cholera presentation (e.g., through cholera interaction with other common acute or chronic diseases,^1^ antibiotic pretreatment that may alter disease course, and different intestinal microbial flora). Because of the common lack of laboratory diagnostic capacity in Africa outside the national capital cities,^11–13^ clinicians and public health staff must rely more on clinical case definitions.

The African Cholera Surveillance Network (Africhol) was launched in 2009 with funding from the Bill & Melinda Gates Foundation. African Cholera Surveillance Network was implemented in 11 countries through 2015 and was designed to assess cholera burden in enhanced surveillance sites and outbreak sites using clinical diagnosis and subsequent laboratory confirmation.^14^ To assess the performance of different cholera case definitions in Africa, we used data from the Africhol surveillance network and outbreak sites to analyze the clinical signs of suspected cholera cases by age group using positive bacterial culture as the gold standard.

## MATERIALS AND METHODS

### Study design.

African Cholera Surveillance Network was implemented in the first countries during 2011 with the collaboration of national ministries of health (MoH). One or more enhanced surveillance areas were identified in each country. Eligibility for inclusion as an Africhol-enhanced surveillance zone was based on meeting all of the following requirements: recent history of high cholera incidence; yearly outbreaks or identification of cholera; hospitals or cholera treatment centers (CTCs) that received most of the patients with cholera; and a laboratory that could perform stool culture for *Vibrio cholerae*. Patients with suspected cholera that were hospitalized or treated in a CTC were included in the analysis. In most sites and most years, cholera was seasonal and consequently CTCs were established only after the first cases during the year were identified; at this point, all patients with suspected cholera usually were referred to these centers.

In addition to surveillance for endemic disease within the enhanced surveillance zones, outbreak investigations were conducted when the national surveillance system reported cholera cases in sites not included in the enhanced surveillance zones. The same methodology (tools, case definition, and training) as in the enhanced surveillance areas was used. Because of budgetary constraints, we focused our efforts on selected outbreaks that occurred in locations not yet studied but having adequate laboratory facilities available. A total of 27 sites in the seven target countries provided data.

### Data collection.

Data were collected on a standardized case report form at each participating treatment center by trained clinical staff used by the National MoH; forms included epidemiological, clinical, behavioral, and laboratory information. Participating treatment centers were all specialized diarrheal disease treatment facilities, either CTCs or diarrheal treatment wards at regional referral hospitals.

We provided standard training to clinical staff at study sites in the evaluation of patients during for the presence of dehydration, difficulty breathing, altered consciousness, dry mucous membranes, and coma. Vomiting, diarrhea, watery stools, rice water stools, and mucous or bloody stools were assessed at the health facility through clinical examination. Patients self-reported symptoms such as nausea, abdominal pain, leg cramps, and number of stools during the 24 hours before admission. Deaths were only ascertained when they occurred in the clinic. All data were entered into the Africhol database.

### Culture confirmation.

In principle, our study aimed to collect rectal swabs from each suspected cholera case. Practical constraints in the field (e.g., clinical staff becoming overwhelmed during the peak of an outbreak) prevented realization of this goal. National public health laboratories in each country performed culture confirmation of suspected cases. In case of work overload during large outbreaks, clinicians were advised to collect rectal swabs from the first 10 patients admitted in the health facility each day, as 10 samples per day was the maximum amount that each laboratory could process. Stool samples were enriched in alkaline peptone water and plated on thiosulfate-citrate-bile-salt-sucrose (TCBS) agar. When characteristic yellow colonies were identified on TCBS and the oxidase test was positive, the patient was considered positive for *V. cholerae*.^15^

### Cholera case definition for the Africhol network.

In areas with known cholera, a suspected case was defined as a patient aged 2 years or more that developed acute watery diarrhea, with or without vomiting. In any particular site, the actual use of the case definitions was unknown even though yearly training was providing to the clinical staff during monitoring visits on sites and questionnaire guidelines were provided. A confirmed cholera case was a person in whom *V. cholerae* was identified by culture and positive oxidase test.

### Participating countries.

Seven countries had more than 35 confirmed cholera cases and were included in the analysis: Côte d’Ivoire, DRC, Guinea, Mozambique, Tanzania, Togo, and Uganda. Prospective surveillance started on different dates for each country. Outside the surveillance zones, cases were identified during outbreak investigations (Table 1). The cut-off point for this analysis was July 2015.

**Table 1:** Participating countries, continuous surveillance sites, and outbreak investigation sites

	Countries	Areas	Health facilities	Start	End
Surveillance	Côte d’Ivoire	Koumassi-Port Bouet-Vridi district	Infectious disease and pediatric departments of Port Bouet and Koumassi Hospitals	August 2011	January 2015
			Vridi Health Center Temporary CTC		
	DRC	Goma and Karimsibi districts	CTCs in General Provincial Hospital	August 2011	July 2015
			Buhimba CTC		
			Kiziba temporary cholera treatment unit		
	Guinea	Five districts of Conakry	Infectious disease and pediatric departments of Donka hospital	July 2011	November 2013
			Additional CTC in Ratoma neighborhood opened during the 2012 epidemic		
	Mozambique	Beira city	Ponta-Gea health center	October 2011	August 2014
			Macurrungo health center		
			Munhava health center		
			Macurrungo health center		
			Beira central hospital		
	Tanzania	Magu district	Magu Hospital	January 2012	March 2013
	–	Mwanza district	Mwanza hospital	–	–
	Togo	Five districts of Lome and Golfe district	Infectious disease and pediatric departments of the Center Hospitalier Universitaire—Be Hospital	June 2011	April 2015
			Other district health centers in which a temporary CTC		
	–	Lake district of the Maritmie region	Infectious disease and pediatric departments of Aneho Hospital	–	–
			Health centers with temporary CTCs		
	Uganda	Butaleja district	Namatela health center	December 2011	June 2015
	–	Manafwa district	Bukigai health center	–	–
	–	Mbale district	Busiu health center	–	–
Outbreak	Côte d’Ivoire	Adiake district	Adiake general hospital	May 2012	October 2012
			Temporary CTC		
	–	Adjame-Plateau-Attecoube, Cocody, East Yopougon, West Yopougon	Adjame-Plateau-Attecoube, Cocody health centers	October 2014	January 2015
			Yopougon general hospital		
	DRC	Kinshasa city	Kingabwa CTC	August 2011	February 2012
			Malaku CTC		
			Massina Cholera treatment unit		
	Guinea	Boffa, Coyah, Dubreka Forecariah, Kindia prefectures	Boffa health center	February 2012	March 2013
			Coyah CTC		
			Dubreka hospital		
			Forecariah CTC		
			Manke CTC		
	Mozambique	Cuamba district	Cuamba CTC	January 2012	February 2012
	–	Montepuez district	Montepuez CTC	February 2012	February 2012
	–	Nampula district	Nampula CTC	February 2013	February 2013
				January 2014	May 2014
	–	Pemba city	Temporary CTC	January 2013	May 2014
	Tanzania	Dar-Es-Salam and Temeke district	Temeke CTC	December 2011	April 2012
	Uganda	Kasese district	Bwera hospital	October 2011	October 2012
			Kayangi health center		
			Kagando hospital		
			Kinyamaseke health center		
			Kitholhu health center		
			Other temporary treatment centers		

CTC = cholera treatment center; DRC = Democratic Republic of Congo.

### Statistical analysis.

Overall and within countries, we analyzed clinical symptoms stratified by the cumulative number of suspected, culture-tested, and culture-confirmed cases. Results are presented by age group and duration of hospitalization. We did not analyze data by site as with 27 different sites case counts were often low, increasing the risk of type 1 errors.

To assess the association between individual symptoms and a positive stool culture for *V. cholerae*, we conducted an analysis limited to patients with a culture result available and aged 1 year or more. Children aged less than 1 year were excluded because of a small sample size (Table 3). All symptom variables were dichotomous. We present unadjusted results from univariate analysis for the association between each individual symptom or sign and culture-confirmed cholera. For each individual symptom or sign that predicted culture positivity on univariate analysis at a significance level of *P* ≤ 0.10, we created a multivariate adjusted model that simultaneously adjusted for gender, age (entered as a linear variable), and country (using DRC as the referent category as a large majority of cases were reported from DRC). We created a separate model for each symptom or sign as symptoms and signs were highly correlated with each other. We considered a multilevel analysis but decided against it because of the small number of countries in our dataset^16^; as noted earlier, instead we opted for stratification to assess the exact degree to which results varied. Missing data represented between 2.4% and 18.6% of cases for individual signs and symptoms (Table 2) and these missing data were evenly distributed over time and across analyzed age groups. Based on this, we decided not to apply multiple imputations for missing data. Data analysis was conducted by Stata version 12 (StataCorp, College Station, TX).

**Table 2:** Repartition of missing data in clinical symptoms and signs of suspected, culture-tested, and culture-confirmed cholera cases in seven countries of the Africhol

Clinical symptom or sign	Suspected = 9,391, *n* (%)	Culture tested = 5,084, *n*′ (%)	Culture confirmed = 1,816, *n*″ (%)
Watery stool	629 (6.7)	121 (2.4)	42 (2.3)
Vomiting	1,248 (13.3)	411 (8.1)	163 (8.9)
Dehydration	1,306 (13.9)	476 (9.4)	185 (10.2)
Rice water stools	914 (9.7)	215 (4.2)	64 (3.5)
Nausea	2,616 (27.9)	917 (18.0)	349 (19.2)
Dry mucous membranes	2,855 (30.4)	944 (18.6)	367 (20.2)
Abdominal pain	1,852 (19.7)	639 (15.6)	250 (13.8)
Leg cramp	2,075 (22.1)	697 (13.7)	259 (14.3)
Mucous stool	1,029 (11.0)	270 (5.3)	84 (4.6)
Altered consciousness	2,298 (24.5)	711 (14.0)	268 (14.8)
Difficulty breathing	2,285 (24.3)	701 (13.8)	268 (14.8)
Coma	2,314 (24.6)	712 (14.0)	270 (14.9)
Bloody stool	969 (10.3)	221 (4.4)	58 (3.2)

Africhol = African Cholera Surveillance Network. *n*, *n*′, *n*″ = total of missing data for symptoms.

Considering the availability of symptoms for each suspect cholera case recorded in the Africhol database, we used three different categories of case definitions:

1. World Health Organization case definition: WHO has two case definitions for cholera, one for areas where disease is not known to be present and one for areas where there is a cholera epidemic. As Africhol sites were selected based, in part, on the known presence of cholera, we assessed only the second, defined as a patient aged more than 5 years old and who develops acute watery diarrhea, with or without vomiting.^17^
2. Ad hoc case definitions developed from combinations of different clinical signs that were identified through multivariate analysis.
3. Country-specific national integrated disease surveillance and response definitions:
  - Côte d’Ivoire, DRC: a patient with severe diarrhea and dehydration or death caused from acute watery diarrhea.
  - Togo: a patient aged five or more years with severe diarrhea and dehydration or death caused from acute watery diarrhea.
  - Mozambique: a patient aged one or more years with acute diarrhea of sudden onset, with or without vomiting or dehydration and regardless of the appearance of the diarrhea.
  - Guinea: a patient aged one or more years with watery diarrhea, with or without vomiting.
  - Tanzania and Uganda: a patient aged two or more years with acute watery diarrhea of sudden onset, with or without vomiting.

Based on culture results as the gold standard, we assessed the sensitivity and specificity of different cholera case definitions by country, identification in an enhanced surveillance zone or outbreak site, and age group. For illustrative purposes, we also present positive and negative predictive values, recognizing that these depend on the a priori likelihood of disease in the study population.

### Ethics.

African Cholera Surveillance Network provided technical and financial resources to national MoHs to support cholera surveillance. Cholera is part of the national public health surveillance through the integrated disease surveillance and response system supported by WHO. The Africhol protocol was approved and implemented by the MoH of each country. The Togolese government further elected to submit the protocol for approval to the national institutional review board (IRB). The remaining countries did not seek IRB approval as they considered that they were conducting epidemic disease surveillance and response covered by national public health laws as an integral part of the public health mandate of the MoH and associated executing agencies.

## RESULTS

A total of 9,391 suspected cholera cases were enrolled in the study from June 2011 to July 2015, with almost half (43.4%) from the DRC, followed by Guinea (20%) and Mozambique (14%) (Table 3). More than 54% of the suspected cases (5,084) had a culture test done, of which 1,816 (35.7%) were confirmed for *V. cholerae*. Goma and its suburbs in DRC represented 60% of culture-confirmed cases. The proportion of men and women was approximately the same within the categories of suspected and culture-tested cases.

**Table 3:** Characteristics of suspected, culture-tested, and culture-confirmed cholera cases in seven countries of the Africhol (*N* = 9,391)

Country	Notification site	Suspected	Culture tested (percentage of suspected)	Culture confirmed (percentage of culture tested)
Côte d’Ivoire	Total	193	136 (70.5)	45 (33.1)
	Surveillance	112	66	18
	Outbreak	81	70	27
DRC	Total	4,074	3,278 (80.5)	1,177 (35.9)
	Surveillance	3,468	2,938	1,041
	Outbreak	606	340	136
Guinea	Total	1,865	261 (14.0)	95 (36.4)
	Surveillance	1,320	143	52
	Outbreak	545	118	43
Mozambique	Total	1,317	430 (32.6)	79 (18.4)
	Surveillance	394	295	2
	Outbreak	923	135	77
Tanzania	Total	151	63 (36.4)	39 (61.9)
	Surveillance	117	8	0
	Outbreak	34	55	39
Togo	Total	630	573 (91.0)	293 (51.1)
	Surveillance	587	540	282
	Outbreak	43	33	11
Uganda	Total	1,161	343 (29.5)	88 (25.7)
	Surveillance	398	159	23
	Outbreak	763	184	65
Sex				
	Female	4,522	2,532 (56.0)	842 (33.3)
	Male	4,543	2,510 (55.2)	955 (38.0)
	Missing	326	42 (12.9)	19 (45.2)
Age group				
	< 1	31	22 (71.0)	3 (13.6)
	1–2	312	226 (72.4)	53 (23.4)
	2–3	461	342 (74.2)	114 (33.3)
	3–4	424	279 (65.8)	122 (43.7)
	4–5	324	234 (72.2)	95 (40.6)
	(5–14)	1,956	1,188 (60.7)	469 (39.5)
	(15–59)	5,133	2,499 (48.7)	874 (35.0)
	> 60	584	266 (45.5)	78 (29.3)
	Unknown	166	28 (17.4)	8 (28.6)
Total	–	9,391	5,084 (54.1)	1,816 (35.7)

Africhol = African Cholera Surveillance Network; DRC = Democratic Republic of Congo.

Within the category of diarrhea, 91.2% of suspected cases and 92.4% of confirmed cases were classified as watery diarrhea, whereas 65.3% of suspected cases and 68.4% of confirmed cases were classified as rice water diarrhea (Table 4). Culture-tested and culture-confirmed cases had approximately the same distribution of symptoms, including a high proportion with watery stools and dehydration, whereas suspected cases were less likely to report any particular symptom. The occurrence of vomiting was different according to age group with 83.5% among children less than age 5 years and 87.0% among persons over age 5 years. Among suspected cases, those < 5 and at least 5 years of age, respectively, had different levels of dry mucous membranes (57.7% versus 50.7%), abdominal pain (55.4% versus 39.2%), and leg cramps (55.6% versus 31.3%) (Table 5). The same difference between age groups was observed for culture confirmed cases except for vomiting (Table 6).

**Table 4:** Clinical symptoms and signs of suspected, culture-tested, and culture-confirmed cholera cases in seven countries of the Africhol; *P* values are shown for associations between symptoms or signs among suspected or culture-tested cases using culture confirmed cases as the referent category

Clinical symptom or sign	Suspected	Culture tested	Culture confirmed
	*n*/*N* (%) (*P* value)	*n*′/*N*′ (%) (*P* value)	*n*″/*N*″ (%)
Watery stool	7,984/8,762 (91.2) (< 0.001)	4,585/4,963 (92.4) (0.18)	1,651/1,774 (93.1)
Vomiting	7,030/8,143 (86.3) (0.003)	3,972/4,673 (85.0) (< 0.001)	1,503/1,653 (90.9)
Dehydration	6,331/6,334 (78.3) (< 0.001)	4,071/4,608 (88.4) (< 0.001)	1,550/1,631 (95.0)
Rice water stools	5,538/8,477 (65.3) (< 0.001)	3,331/4,869 (68.4) (< 0.001)	1,327/1,752 (75.7)
Nausea	4,864/6,775 (71.8) (< 0.001)	3,228/4,167 (77.5) (< 0.001)	1,193/1,467 (81.3)
Dry mucous membranes	3,654/6,536 (55.9) (< 0.001)	2,716/4,160 (65.6) (< 0.001)	1,037/1,449 (71.6)
Abdominal pain	3,953/7,316 (52.4) (< 0.001)	2,471/4,445 (55.6) (0.55)	861/1,566 (55.0)
Leg cramp	3,758/7,316 (51.4) (< 0.001)	2,312/4,387 (52.7) (< 0.001)	928/1,557 (59.6)
Mucous stool	461/8,362 (5.5) (< 0.001)	340/4,814 (7.1) (0.969)	122/1,732 (7.0)
Altered consciousness	354/7,093 (5.0) (0.54)	194/4,373 (4.4) (0.019)	84/1,548 (5.4)
Difficulty breathing	262/7,106 (3.7) (0.084)	148/4,383 (3.4) (0.064)	63/1,551 (4.0)
Coma	81/7,077 (1.1) (0.11)	54/4,372 (1.2) (0.16)	24/1,546 (1.6)
Bloody stool	48/8,422 (0.6) (0.055)	31/4,863 (0.6) (0.004)	2/1,758 (0.1)

Africhol = African Cholera Surveillance Network. *n*, *n*′, *n*″ = total of presence of symptoms/*N*, *N*′, *N*″ = total of the cases with data available

**Table 5:** Distribution of clinical symptoms and signs by age group for all suspected cholera cases (*N* = 9,230)

	Watery stool	10+ stools in 24 hours	Vomiting	Dehydration	Rice water stools	Nausea	Dry mucous membra-nes	Abdominal pain	Leg cramp	Mucous stool	Altered conscious-ness	Difficulty breathing	Coma	Bloody stool
Age < 5 years	1,357	508	1,171	1,193	1,004	874	633	500	391	83	52	44	18	3
Percentage	92.1	38.6	83.5	85.6	69.2	75.3	50.7	39.2	31.3	5.8	4.0	3.4	1.4	0.2
Age ≥ 5 years	6,523	2,060	5,767	5,098	4,465	3,960	3,003	3,419	3,323	374	301	214	63	45
Percentage	91.1	35.4	87.0	77.4	64.6	71.4	57.7	55.4	55.6	5.5	5.3	3.7	1.1	0.7
*P* value	0.185	0.028	0.001	< 0.001	0.001	0.007	< 0.001	< 0.001	< 0.001	0.705	0.064	0.541	0.372	0.041

**Table 6:** Distribution of clinical symptoms and signs by age group for confirmed cholera cases (*N* = 1,808)

	Watery stool	10+ stools in 24 hours	Vomiting	Dehydration	Rice water stools	Nausea	Dry mucous membra-nes	Abdominal pain	Leg cramp	Mucous stool	Altered conscious-ness	Difficulty breathing	Coma	Bloody stool
Age < 5 years	361	172	332	351	299	255	219	141	115	31	16	7	7	0
Percentage	94.8	48.3	90.7	97.0	79.5	84.2	64.6	42.5	35.0	8.3	4.5	2.0	2.0	0.0
Age ≥ 5 years	1,286	562	1,167	1,195	1,025	936	816	716	812	91	68	56	17	2
Percentage	92.7	47.4	91.0	94.5	74.8	80.6	73.65	58.2	66.3	6.7	5.7	4.1	1.4	0.2
*P* value	0.164	0.748	0.884	0.054	0.056	0.151	0.001	< 0.001	< 0.001	0.304	0.386	0.025	0.455	0.458

Of all suspected cases, those hospitalized for more than 1 day had more frequent stooling, (i.e., > 10 stools in the 24 hours before admission), nausea, vomiting, dehydration, dry mucous membranes, abdominal pain, and leg cramps than those hospitalized less than 1 day (Table 7). By contrast, among confirmed cases, only those hospitalized for more than 3 days were more frequently diagnosed with dehydration (91.7% versus 87.9%), as well as having between 10 and 25 stools in the last 24 hours (49.8% versus 28.1%), nausea (83.4% versus 56.7%), and dry mucous membranes (76.6% versus 50.0%) compared with those hospitalized less than a day (Table 8).

**Table 7:** Distribution of clinical symptoms and signs by duration of hospitalization for all suspected cases (*N* = 5,768)

	Watery stool	10+ stools in 24 hours	Vomiting	Dehydration	Rice water stools	Nausea	Dry mucous mem-branes	Abdominal pain	Leg cramp	Mucous stool	Altered consciousness	Difficulty breathing	Coma	Bloody stool
Hosp. less than 1 day	155	28	133	93	122	73	63	59	48	6	8	3	1	4
Percentage*	90.1	18.5	82.1	57.4	71.4	55.3	44.1	37.1	31.2	3.5	5.3	1.9	0.7	0.6
Hosp. 1 day	934	240	800	634	733	555	428	415	402	67	31	27	6	9
Percentage	94.3†	26.0†	84.1	67.1†	75.2	66.6†	48.6	45.3	43.6†	6.9	3.4	2.9	0.7	0.9
Hosp. 2 days	1,549	551	1,426	1,220	1,251	1,018	848	776	808	88	58	42	13	6
Percentage	94.7†	35.8†	89.5†	77.1†	77.7	73.1†	57.5†	51.7†	53.1†	5.5	3.8	2.8	0.9	0.4
Hosp. 3 days+	2,785	1,064	2,574	2,364	2,200	1,901	1,516	1,478	1,514	144	153	88	33	14
Percentage	94.9†	41.4†	91.6†	84.4†	76.1	77.4†	63.6†	55.5†	58.7†	5.0	5.9	3.4	1.3	0.5

* Referral category.

† *P* value < 0.05 for an association between the symptom and likelihood of one or more hospital days (i.e., odds ratio > 1).

**Table 8:** Distribution of clinical symptoms and signs by duration of hospitalization for culture-confirmed cholera cases (*N* = 1,253)

	Watery stool	10+ stools in 24 hours	Vomiting	Dehydration	Rice water stools	Nausea	Dry mucous membranes	Abdominal pain	Leg cramp	Mucous stool	Altered consciousness	Difficulty breathing	Coma	Bloody stool
Hosp. less than 1 day	33	9	29	29	31	17	15	15	20	3	7	2	1	0
Percentage*	100.0	28.1	90.6	87.9	94.0	56.7	50.0	45.5	69.0	9.1	23.3	6.5	3.3	0.0
Hosp. 1 day	139	59	117	128	115	102	89	77	65	77	7	4	2	0
Percentage	99.3	44.7†	83.6	92.8	82.1	80.3†	67.4	56.2	47.5‡	5.1	5.2‡	2.9	1.5	0.0
Hosp. 2 days	313	141	295	288	259	255	209	176	172	26	13	11	3	0
Percentage	98.1	46.2†	93.7	92.6	81.7	86.7†	71.6†	58.5	57.1	8.4	4.3‡	3.6	1.0	0.0
Hosp. 3 days+	739	349	696	713	622	571	521	406	427	44	40	31	9	2
Percentage	97.5	49.8†	93.3	97.1†	82.5	83.4†	76.6†	56.8	60.4	5.9	5.1‡	4.1	1.3	0.3

* Referral category.

† *P* value < 0.05 for an association between the symptom and likelihood of one or more hospital days.

‡ *P* value < 0.05 for an association between the symptom and likelihood of less than one hospital day (i.e., odds ratio < 1).

Rice water stools, vomiting, nausea, severe dehydration, dry mucous membranes, leg cramps, and altered consciousness predicted positive stool culture in univariate analysis. Bloody stools predicted a negative stool culture, although this symptom was reported for only 31 patients (Table 9). When adjusting for gender, age, and country, all of these symptoms remained associated with culture positivity.

**Table 9:** Distribution of collected samples and culture confirmation status in seven member countries of the Africhol

			Culture	Univariate		Multivariate	
Symptoms		*N*	Positive *N* (%)	OR	95% CI	OR	95% CI
Watery stool	No	378	123 (32.5)	Ref.	–	–	–
	Yes	4,585	1,651 (36.0)	1.17	0.93–1.46	–	–
Number of stools in 24 hours	0–9	2,382	814 (34.1)	Ref.	–	Ref.	–
	10–25	1,951	735 (37.6)	1.17	1.03–1.32	1.17	1.02–1.34
Rice water stools	No	1,538	425 (27.6)	Ref.	–	Ref.	–
	Yes	3,331	1,327 (39.8)	1.73	1.52–1.98	1.86	1.60–2.17
Mucous stool	No	4,474	1,610 (36.0)	Ref.	–	–	–
	Yes	340	122 (35.9)	0.99	0.79–1.25	–	–
Bloody stool	No	4,832	1,756 (36.3)	Ref.	–	Ref.	–
	Yes	31	2 (6.5)	0.12	0.03–0.51	0.08	0.02–0.34
Nausea	No	939	274 (29.2)	Ref.	–	Ref.	–
	Yes	3,228	1,193 (37.0)	1.42	1.22–1.67	1.34	1.13–1.59
Vomiting	No	701	150 (21.4)	Ref.	–	Ref.	–
	Yes	3,972	1,503 (37.8)	2.23	1.85–2.71	2.00	1.64–2.46
Dry mucus membranes	No	1,424	412 (28.9)	Ref.	–	Ref.	–
	Yes	2,716	1,037 (38.2)	1.51	1.32–1.74	1.57	1.35–1.82
Dehydration	No	537	81 (15.1)	Ref.	–	Ref.	–
	Yes	4,071	1,550 (38.1)	3.46	2.71–4.42	4.98	3.75–6.62
Abdominal pain	No	1,974	705 (35.7)	Ref.	–	–	–
	Yes	2,471	861 (34.8)	0.96	0.85–1.09	–	–
Leg cramps	No	2,075	629 (30.3)	Ref.	–	Ref.	–
	Yes	2,312	928 (40.1)	1.45	1.27–1.64	1.59	1.39–1.82
Difficulty breathing	No	4,235	1,488 (35.1)	Ref.	–	–	–
	Yes	148	63 (42.6)	1.37	0.98–1.91	–	–
Altered consciousness	No	4,179	1,464 (35.0)	Ref.	–	Ref.	–
	Yes	194	84 (43.3)	1.42	1.06–1.89	1.41	1.04–1.92
Coma	No	4,318	1,522 (35.3)	Ref.	–	–	–
	Yes	54	24 (44.4)	1.43	0.86–2.52	–	–

Africhol = African Cholera Surveillance Network; CI = confidence interval; OR = odds ratio; Ref. = referent category. A separate multivariate model was created for each symptom that predicted culture positivity on univariate analysis by simultaneously adjusting from gender, age (for those at least age 1 year), and country.

For all countries combined, the WHO case definition had a high sensitivity (92.7%; 95% CI: 91.2–94.0) but low specificity (8.1%; 95% CI: 7.1–9.3) among persons aged at least 5 years. For those aged 1–4 years, the WHO case definition had a sensitivity of 94.0% (95% CI: 91.1–96.1) and specificity of 6.1% (95% CI: 4.4–8.1) (Tables 10 and 11). A case definition combining watery stools or rice water stools with either dehydration or vomiting substantially improved specificity without lowering sensitivity. The definition that achieved the highest specificity was rice water stools associated with the occurrence of less than 10 stools in the last 24 hours: 70.2% for cases aged more than 5 years and 65.1% for cases aged 1–4 years.

**Table 10:** Sensitivity, specificity, PPV, and NPV for tested cases aged more than 5 years

Case definition	Sensitivity % (95% CI)	Specificity % (95% CI)	PPV % (95% CI)	NPV % (95% CI)
World Health Organization epidemic cholera definition	92.7 (91.2–94.0)	8.1 (7.1–9.3)	36.1 (34.5–37.7)	66.6 (60.9–71.9)
Watery stool and dehydration	92.1 (90.5–93.6)	17.3 (15.8–18.9)	38.2 (36.5–40.0)	79.9 (76.1–83.3)
Watery stool and vomiting	88.8 (86.9–90.5)	21.0 (19.3–22.7)	38.3 (36.5–40.1)	77.2 (73.7–80.4)
Watery stool and ≤ 9 stools in last 24 hours	51.2 (48.3–54.1)	46.5 (44.4–48.7)	34.5 (32.3–36.8)	63.4 (61.0–65.8)
Watery stool and (10–25) stools in last 24 hours	47.0 (44.1–49.9)	57.3 (55.2–59.4)	37.7 (35.2–40.2)	66.3 (64.1–68.4)
Watery stool and dry mucous membranes	72.2 (69.5–74.8)	34.1 (32.0–36.2)	37.2 (35.2–39.3)	69.4 (66.4–72.2)
Watery stool and dehydration and vomiting	84.9 (82.8–86.8)	26.6 (24.8–28.5)	39.3 (37.4–41.1)	75.9 (72.7–78.9)
Watery stool and dehydration and ≤ 9 stools in last 24 hours	46.7 (43.8–49.6)	58.5 (56.3–60.6)	38.7 (36.1–41.3)	66.2 (64.0–68.3)
Watery stool and dehydration and (10–25) stools in last 24 hours	46.7 (43.8–49.6)	58.3 (56.2–60.4)	37.9 (35.4–40.5)	66.7 (64.5–68.9)
Watery stool and (dehydration or vomiting)	96.7 (95.6–97.6)	10.7 (9.4–12.1)	37.7 (36.0–39.4)	85.3 (80.6–89.2)
Watery stool and (dehydration or rice water stools)	95.9 (94.7–96.9)	14.6 (13.1–16.1)	38.6 (36.9–40.4)	86.4 (82.5–89.7)
Watery stool and (dehydration or rice water stools or vomiting)	97.2 (96.1–98.0)	10.0 (8.8–11.3)	37.8 (36.1–39.6)	86.2 (81.4–90.2)
Watery stool and vomiting and dry mucous membranes	68.6 (65.8–71.3)	40.1 (38.0–42.3)	38.3 (36.2–40.5)	70.2 (67.5–72.8)
Rice water stools	74.8 (72.4–77.0)	36.6 (34.7–38.6)	40.1 (38.2–42.0)	71.9 (69.3–74.4)
Rice water stools and dehydration	75.6 (73.1–77.9)	36.1 (34.2–38.2)	39.8 (37.8–41.8)	72.6 (69.9–75.2)
Rice water stools and vomiting	73.4 (70.9–75.8)	41.2 (39.2–43.3)	41.1 (39.1–43.2)	73.5 (70.9–75.9)
Rice water stools and ≤ 9 stools in last 24 hours	40.2 (37.4–43.1)	70.2 (68.2–72.1)	42.7 (39.8–45.7)	68.0 (66.0–69.9)
Rice water stools and (10–25) stools in last 24 hours	40.3 (37.5–43.2)	62.9 (60.8–65.0)	37.5 (34.9–40.3)	65.6 (63.5–67.6)
Rice water stools and dry mucous membranes	60.9 (58.0–63.8)	46.0 (43.9–48.2)	38.1 (35.8–40.4)	68.4 (65.9–70.9)
Rice water stools and dehydration and vomiting	70.8 (68.2–73.4)	42.5 (40.4–44.6)	40.9 (38.8–43.0)	72.2 (69.7–74.6)
Rice water stools and dehydration and ≤ 9 stools in last 24 hours	38.5 (35.6–41.4)	69.4 (67.2–71.5)	43.0 (40.0–46.2)	65.2 (63.1–67.3)
Rice water stools and dehydration and (10–25) stools in last 24 hours	39.0 (36.2–41.9)	66.0 (64.0–68.0)	37.3 (34.6–40.1)	67.6 (65.6–69.5)
Rice water stools and vomiting and dry mucous membranes	58.1 (55.2–61.1)	51.1 (48.9–53.3)	39.3 (37.0–41.8)	69.1 (66.7–71.4)

CI = confidence interval; NPV = negative predictive value; PPV = positive predictive value.

**Table 11:** Sensitivity, specificity, PPV, and NPV for tested cases aged between 1 and 4 years

Case definition	Sensitivity % (95% CI)	Specificity % (95% CI)	PPV % (95% CI)	NPV % (95% CI)
World Health Organization epidemic cholera definition	94.0 (91.1–96.1)	6.1 (4.4–8.1)	36.1 (33.1–39.2)	64.1 (51.1–75.7)
Watery stool and dehydration	95.0 (92.2–97.0)	19.0 (16.1–22.2)	39.3 (36.1–42.7)	87.3 (80.7–92.3)
Watery stool and vomiting	87.9 (84.2–91.1)	23.3 (20.1–26.7)	39.1 (35.7–42.5)	77.6 (71.1–83.2)
Watery stool and ≤ 9 stools in the last 24 hours	50.4 (45.1–55.7)	44.4 (40.5–48.4)	33.7 (29.7–37.9)	61.5 (56.9–66.0)
Watery stool and (10–25) stools in the last 24 hours	47.9 (42.6–53.2)	59.4 (55.5–63.3)	39.8 (35.1–44.6)	67.0 (63.0–70.9)
Watery stool and dry mucous membranes	63.8 (58.5–68.9)	57.4 (53.4–61.3)	45.3 (40.8–49.9)	74.2 (70.0–78.0)
Watery stool and dehydration and vomiting	85.5 (81.4–89.0)	28.8 (25.3–32.5)	40.1 (36.6–43.7)	78.1 (72.2–83.2)
Watery stool and dehydration and ≤ 9 stools in the last 24 hours	47.9 (42.5–53.2)	58.9 (54.9–62.7)	39.3 (34.6–44.1)	67.0 (62.9–70.9)
Watery stool and dehydration and (10–25) stools in the last 24 hours	47.9 (42.5–53.2)	59.1 (55.1–63.0)	39.9 (35.1–44.7)	66.7 (62.5–70.6)
Watery stool and (dehydration or vomiting)	97.8 (95.6–99.0)	12.6 (10.2–15.5)	38.5 (35.3–41.7)	91.0 (83.1–96.0)
Watery stool and (dehydration or rice water stools)	97.2 (94.9–98.6)	18.4 (15.4–21.6)	39.8 (36.5–43.1)	92.2 (86.1–96.2)
Watery stool and (dehydration or rice water stools or vomiting)	98.0 (96.0–99.2)	12.5 (10.0–15.3)	38.6 (35.4–41.8)	91.9 (83.9–96.7)
Watery stool and vomiting and dry mucous membranes	57.5 (52.1–62.8)	61.7 (57.7–65.6)	45.6 (40.8–50.4)	72.3 (68.2–76.1)
Rice water stools	78.8 (74.4–82.8)	30.0 (26.6–33.7)	38.9 (35.4–42.5)	71.5 (65.9–76.7)
Rice water stools and dehydration	80.2 (75.7–84.2)	29.8 (26.3–33.5)	38.7 (35.2–42.3)	73.1 (67.3–78.4)
Rice water stools and vomiting	73.4 (68.5–77.9)	35.9 (32.2–39.7)	39.0 (35.3–42.8)	70.7 (65.5–75.6)
Rice water stools and ≤ 9 stools in the last 24 hours	39.5 (34.3–44.8)	65.1 (61.2–68.8)	38.3 (33.1–43.7)	66.6 (62.6–70.4)
Rice water stools and (10–25) stools in the last 24 hours	44.3 (39.1–49.7)	62.7 (58.8–66.5)	40.0 (35.1–45.1)	66.7 (62.8–70.5)
Rice water stools and dry mucous membranes	54.5 (49.0–60.0)	61.0 (57.1–64.9)	43.1 (38.3–48.0)	71.3 (67.2–75.2)
Rice water stools and dehydration and vomiting	71.8 (66.8–76.5)	36.3 (32.5–40.1)	38.6 (34.8–42.4)	69.8 (64.5–74.7)
Rice water stools and dehydration and ≤ 9 stools in the last 24 hours	40.3 (34.9–45.9)	64.7 (60.7–68.5)	38.3 (33.1–43.7)	66.6 (62.6–70.4)
Rice water stools and dehydration and (10–25) stools in the last 24 hours	41.5 (36.4–46.7)	63.8 (59.9–67.5)	39.8 (34.9–44.9)	65.3 (61.4–69.1)
Rice water stools and vomiting and dry mucous membranes	47.9 (42.5–53.4)	64.8 (60.8–68.6)	43.2 (38.1–48.4)	69.0 (65.0–72.8)

CI = confidence interval; NPV = negative predictive value; PPV = positive predictive value.

Data were similar when stratified by country, although some modest and occasional differences were found (Supplemental Tables 1–11). For example, the WHO case definition’s sensitivity was lower in Mozambique (64.5%; 95% CI: 52.7–75.1) than that in other countries (from 87.5% to 98.9%). Similarly, the WHO case definition had a lower sensitivity in outbreak sites versus in the enhanced surveillance zones (75.8% versus 97.8%) (Supplemental Tables 12–15). The WHO case definition sensitivity and specificity did not change by individual age year for children between 1 and 4 years old (Supplemental Table 16). Country-specific case definitions had variable sensitivity and specificity, with sensitivity varying from 46.5% in Côte d’Ivoire to 100% in Mozambique and specificity varying from 4.1% in Mozambique and Uganda to 63.0% in Togo (Supplemental Table 17).

## DISCUSSION

In using data from 27 sites located in seven African countries, we found that the WHO case definition for cholera had high sensitivity and low specificity overall and among all subgroups. This is likely because almost all true cholera cases typically had acute watery diarrhea but most patients with acute watery diarrhea did not have cholera, even in outbreak sites. Use of a highly sensitive definition facilitates the timely detection of outbreaks and may be appropriate given the potential for cholera to rapidly cause large outbreaks or national epidemics.^18–21^ However, the lack of specificity may lead to excessive interventions or inappropriate distribution of resources. Adding the presence of other symptoms to the WHO case definition used during outbreaks—such as dehydration, vomiting, or rice water stools—substantially increased specificity with only a minimal decrease in sensitivity. Other studies of cholera have shown that the same clinical signs were associated with culture positivity among suspected cholera cases.^10^ For example, in Bangladesh, diarrhea, vomiting, and dehydration were more frequent in culture-positive cases.^22^ As in Haiti,^23^ we found that persons with longer hospitalization periods were more likely to have dehydration and signs of dehydration compared with persons hospitalized less than a day.

The WHO cholera case definition excludes children aged less than 5 years old to avoid expending resources on investigation of routine infant diarrhea.^1^ This policy is based on the assumption that a much higher percentage of early childhood diarrhea will result from causes other than cholera when compared with older persons. However, this assumption may be incorrect. In our study, the WHO epidemic cholera case definition had low specificity and was similar for persons aged between 1 and 4 years and 5+ years of age, a finding that occurred across all settings and within individual year ages among children aged 1–4 years. We also found that 21% of all confirmed cases were aged 1–4 years, which is in agreement with data from other studies in cholera endemic areas that have found cholera is a primary cause of diarrhea in this age group.^24^ These data suggest that the WHO case definition should incorporate children aged between 1 and 4 years, as has been done with many national case definitions.

Our study had several limitations. We did not have complete knowledge of why persons did or did not have a stool culture performed, nor did we have complete knowledge of the source populations from which our cases derived. This limitation could reduce the ability to apply our results to other settings. Nevertheless, our data were derived from seven different countries and results were similar across sites. Moreover, our data reflect results from real-world settings where clinical and MoH staff have limited data availability, including on source populations and reasons for the lack of stool culture.

Because of laboratory limitations in African field settings, we used culture confirmation as the gold standard despite a reported sensitivity of 66%.^25^ Consequently, a substantial portion of cases reported as culture negative may have had true cholera.^14^ This limitation should be addressed in future studies by use of a combination of several more sensitive testing methods.^26^

Differing levels of antibiotic pretreatment might impact subsequent case definition performance. To address this issue, we originally had planned to collect data on antibiotic use; however, these data were usually not entered on the data collection form and where data were included were incomplete (for example, the name of the medication or whether it was an antibiotic). Future studies might address this issue by testing urine antibiotic activity. Ideally, with further resources and funding available, we would have chosen to test the clinical definitions also against polymerase chain reaction to increase sensitivity followed by determination of the optimal clinical definition across all methods. We would encourage funders and researchers to consider investing in such a study.

Our study was conducted in limited areas and thus may not be representative of the remainder of participating countries or of nonparticipating countries, although the relative consistency of results suggests that this was not a major limitation. During cholera outbreaks, clinical and public health staff can be overwhelmed and thus may not complete case report forms fully and rectal swabs could not be collected for all patients; moreover, it is possible that staff focused on symptoms they thought were associated with cholera.

To our knowledge, this is the only recent study specifically on cholera symptoms in Africa. Our results suggest that the WHO case definition could be made more specific without a substantial decrease in sensitivity by adding one or more symptoms. However, the observed heterogeneity in the adherence of clinical staff to an established case definition across countries raises concerns of whether a more complex and hence specific case definition could be reliably implemented at least in the sites where we worked.

Furthermore, our results support inclusion of children aged between 1 and 4 years old in the case definition when cholera is known to be present. Besides improving estimates of disease burden, this would have the benefit of sensitizing clinicians, public-health workers, and stakeholders to the risk of cholera among young children, allowing for the mobilization of additional prevention efforts. Future studies could refine our findings primarily by using more sensitive methods for cholera confirmation.

## Supplementary Material

Supplemental Tables.
