# Supplementary material for: Sensitivity, Specificity, and Public-Health Utility of Clinical Case Definitions Based on the Signs and Symptoms of Cholera in Africa
Source: Am J Trop Med Hyg. 2018 Feb 26;98(4):1021–30. doi: 10.4269/ajtmh.16-0523 (PMC5928804; doi:10.4269/ajtmh.16-0523)
Supplement: Supplementary file 1 [file tpmd160523.SD1.pdf]

Supplemental Table 1

Sensitivity, specificity, PPV, and NPV for tested cases aged more than 5 years in Côte d'Ivoire

Case definition	Sensitivity % (95% CI)	Specificity % (95% CI)	PPV % (95% CI)	NPV % (95% CI)
World Health Organization epidemic cholera definition	87.5 (73.2–95.8)	8.8 (3.6–17.2)	32.4 (23.7–42.1)	58.3 (27.7–84.8)
Watery stool and dehydration	72.0 (50.6–87.9)	37.0 (24.3–51.3)	34.6 (22.0–49.1)	74.1 (53.7–88.9)
Watery stool and vomiting	82.1 (63.1–93.9)	16.9 (8.4–29.0)	31.9 (21.4–44.0)	66.7 (38.4–88.2)
Watery stool and ≤ 9 stools in the last 24 hours	47.1 (23.0–72.2)	58.7 (43.2–73.0)	29.6 (13.8–50.2)	75.0 (57.8–87.9)
Watery stool and (10–25) stools in the last 24 hours	52.9 (27.8–77.0)	43.5 (28.9–58.9)	25.7 (12.5–43.3)	71.4 (51.3–86.8)
Watery stool and dry mucous membranes	30.8 (9.1–61.4)	59.5 (42.1–75.2)	21.1 (6.1–45.6)	71.0 (52.0–85.8)
Watery stool and dehydration and vomiting	73.9 (51.6–89.8)	44.4 (30.9–58.6)	36.2 (22.7–51.5)	80.0 (61.4–92.3)
Watery stool and dehydration and ≤ 9 stools in the last 24 hours	36.4 (10.9–69.2)	83.3 (62.6–95.3)	50.0 (15.7–84.3)	74.1 (53.7–88.9)
Watery stool and dehydration and (10–25) stools in the last 24 hours	41.2 (18.4–67.1)	63.3 (49.9–75.4)	24.1 (10.3–43.5)	79.2 (65.0–89.5)
Watery stool and (dehydration or vomiting)	91.7 (73.0–99.0)	9.3 (3.1–20.3)	31.0 (20.5–43.1)	71.4 (29.0–96.3)
Watery stool and (dehydration or rice water stools)	84.0 (63.9–95.5)	25.5 (14.3–39.6)	35.6 (23.6–49.1)	76.5 (50.1–93.2)
Watery stool and (dehydration or rice water stools or vomiting)	91.7 (73.0–99.0)	9.8 (3.3–21.4)	32.4 (21.5–44.8)	71.4 (29.0–96.3)
Watery stool and vomiting and dry mucous membranes	30.8 (9.1–61.4)	59.5 (42.1–75.2)	21.1 (6.1–45.6)	71.0 (52.0–85.8)
Rice water stools	28.2 (15.0–44.9)	74.3 (62.8–83.8)	36.7 (19.9–56.1)	66.3 (55.1–76.3)
Rice water stools and dehydration	20.0 (6.8–40.7)	77.4 (63.8–87.7)	29.4 (10.3–56.0)	67.2 (54.0–78.7)
Rice water stools and vomiting	28.6 (13.2–48.7)	71.9 (58.5–83.0)	33.3 (15.6–55.3)	67.2 (54.0–78.7)
Rice water stools and ≤ 9 stools in the last 24 hours	5.9 (0.1–28.7)	95.5 (84.5–99.4)	33.3 (0.8–90.6)	72.4 (59.1–83.3)
Rice water stools and (10–25) stools in the last 24 hours	29.4 (10.3–56.0)	79.5 (64.7–90.2)	35.7 (12.8–64.9)	74.5 (59.7–86.1)
Rice water stools and dry mucous membranes	15.4 (1.9–45.4)	83.8 (68.0–93.8)	25.0 (3.2–65.1)	73.8 (58.0–86.1)
Rice water stools and dehydration and vomiting	20.8 (7.1–42.2)	81.1 (68.0–90.6)	33.3 (11.8–61.6)	69.4 (56.3–80.4)
Rice water stools and dehydration and ≤ 9 stools in the last 24 hours	8.3 (0.2–38.5)	100.0 (89.1–100.0)	100.0 (2.5–100.0)	74.4 (58.8–86.5)
Rice water stools and dehydration and (10–25) stools in the last 24 hours	18.8 (4.0–45.6)	83.3 (69.8–92.5)	27.3 (6.0–61.0)	75.5 (61.7–86.2)
Rice water stools and vomiting and dry mucous membranes	15.4 (1.9–45.4)	83.8 (68.0–93.8)	25.0 (3.2–65.1)	73.8 (58.0–86.1)

CI = confidence interval; NPV = negative predictive value; PPV = positive predictive value.

Supplemental Table 2

Sensitivity, specificity, PPV, and NPV for tested cases aged more than 5 years in DRC

Case definition	Sensitivity % (95% CI)	Specificity % (95% CI)	PPV % (95% CI)	NPV % (95% CI)
World Health Organization epidemic cholera definition	92.6 (90.5–94.3)	5.9 (4.8–7.2)	34.0 (32.0–36.0)	60.3 (52.0–68.1)
Watery stool and dehydration	95.9 (94.2–97.2)	6.1 (5.0–7.5)	34.3 (32.3–36.3)	74.6 (65.9–82.0)
Watery stool and vomiting	90.2 (87.9–92.2)	14.8 (13.0–16.7)	34.9 (32.8–37.0)	74.8 (69.5–79.7)
Watery stool and ≤ 9 stools in the last 24 hours	43.9 (40.3–47.6)	57.8 (55.2–60.3)	34.6 (31.6–37.8)	66.9 (64.2–69.5)
Watery stool and (10–25) stools in the last 24 hours	53.8 (50.1–57.4)	45.2 (42.6–47.8)	33.4 (30.7–36.1)	65.7 (62.7–68.7)
Watery stool and dry mucous membranes	78.8 (75.6–81.7)	22.4 (20.2–24.6)	34.3 (32.0–36.6)	67.2 (62.8–71.4)
Watery stool and dehydration and vomiting	88.8 (86.4–91.0)	15.8 (14.0–17.8)	35.0 (32.9–37.2)	73.5 (68.3–78.3)
Watery stool and dehydration and ≤ 9 stools in the last 24 hours	47.9 (44.0–51.8)	52.9 (50.0–55.8)	35.6 (32.4–38.9)	65.2 (62.1–68.2)
Watery stool and dehydration and (10–25) stools in the last 24 hours	48.3 (44.8–51.8)	52.1 (49.7–54.6)	33.3 (30.6–36.1)	67.1 (64.5–69.7)
Watery stool and (dehydration or vomiting)	97.7 (96.4–98.7)	4.0 (3.1–5.1)	34.2 (32.2–36.3)	77.6 (66.6–86.4)
Watery stool and (dehydration or rice water stools)	97.2 (95.8–98.3)	4.2 (3.2–5.4)	34.2 (32.2–36.3)	74.7 (64.0–83.6)
Watery stool and (dehydration or rice water stools or vomiting)	97.9 (96.6–98.8)	3.5 (2.6–4.6)	34.2 (32.2–36.3)	76.1 (64.1–85.7)
Watery stool and vomiting and dry mucous membranes	74.5 (71.1–77.6)	29.9 (27.5–32.3)	35.3 (32.9–37.7)	69.5 (65.9–73.1)
Rice water stools	80.6 (77.7–83.3)	17.6 (15.7–19.6)	33.9 (31.8–36.1)	63.4 (58.6–68.0)
Rice water stools and dehydration	83.8 (81.0–86.4)	17.2 (15.3–19.2)	34.1 (32.0–36.4)	67.5 (62.5–72.2)
Rice water stools and vomiting	79.2 (76.1–82.0)	25.1 (22.9–27.3)	35.0 (32.7–37.3)	70.3 (66.2–74.1)
Rice water stools and ≤ 9 stools in the last 24 hours	36.4 (33.0–40.0)	65.0 (62.5–67.5)	34.8 (31.4–38.3)	66.7 (64.1–69.1)
Rice water stools and (10–25) stools in the last 24 hours	48.8 (45.1–52.5)	49.6 (47.0–52.2)	33.1 (30.3–36.0)	65.4 (62.5–68.2)
Rice water stools and dry mucous membranes	69.9 (66.4–73.2)	30.1 (27.7–32.6)	34.0 (31.6–36.6)	66.0 (62.2–69.6)
Rice water stools and dehydration and vomiting	78.3 (75.2–81.2)	25.5 (23.3–27.8)	35.1 (32.8–37.4)	69.6 (65.5–73.5)
Rice water stools and dehydration and ≤ 9 stools in the last 24 hours	40.9 (37.1–44.9)	69.5 (56.6–62.3)	35.5 (32.0–39.1)	64.9 (61.9–67.8)
Rice water stools and dehydration and (10–25) stools in the last 24 hours	43.0 (39.6–46.5)	56.7 (54.3–59.1)	33.0 (30.2–35.9)	66.7 (64.2–69.2)
Rice water stools and vomiting and dry mucous membranes	66.6 (63.0–70.0)	36.7 (34.2–39.3)	35.1 (32.7–37.8)	68.0 (64.5–71.3)

CI = confidence interval; DRC = Democratic Republic of Congo; NPV = negative predictive value; PPV = positive predictive value.

Supplemental Table 3

Sensitivity, specificity, PPV, and NPV for tested cases aged between 1 and 4 years in DRC

Case definition	Sensitivity % (95% CI)	Specificity % (95% CI)	PPV % (95% CI)	NPV % (95% CI)
World Health Organization epidemic cholera definition	94.6 (91.6–96.8)	5.0 (3.3–7.3)	39.2 (35.8–42.7)	59.1 (43.2–73.7)
Watery stool and dehydration	95.9 (93.2–97.8)	5.1 (3.4–7.5)	39.1 (35.7–42.6)	66.7 (49.8–80.9)
Watery stool and vomiting	88.2 (84.2–91.5)	15.3 (12.3–18.8)	39.7 (36.1–43.4)	67.2 (57.9–75.7)
Watery stool and ≤ 9 stools in the last 24 hours	48.0 (42.4–53.6)	53.5 (49.0–58.0)	39.8 (34.9–44.9)	61.6 (56.8–66.2)
Watery stool and (10–25) stools in the last 24 hours	50.5 (44.8–56.1)	38.8 (34.1–43.7)	39.3 (34.5–44.2)	50.0 (44.4–55.6)
Watery stool and dry mucous membranes	65.0 (59.4–70.3)	50.4 (45.9–54.9)	45.5 (40.8–50.3)	69.3 (64.2–74.1)
Watery stool and dehydration and vomiting	86.5 (83.0–90.6)	15.6 (12.5–19.0)	39.5 (35.8–43.2)	64.5 (55.2–73.0)
Watery stool and dehydration and ≤ 9 stools in the last 24 hours	48.2 (42.4–54.0)	52.1 (47.5–56.7)	39.5 (34.4–44.6)	60.8 (55.9–65.7)
Watery stool and dehydration and (10–25) stools in the last 24 hours	48.3 (42.8–53.9)	52.0 (47.5–56.4)	39.3 (34.5–44.2)	61.0 (56.3–65.6)
Watery stool and (dehydration or vomiting)	98.1 (96.0–99.3)	4.4 (2.8–6.6)	39.6 (36.2–43.1)	78.6 (59.0–91.7)
Watery stool and (dehydration or rice water stools)	97.8 (95.5–99.1)	4.4 (2.8–6.6)	39.4 (36.0–42.9)	75.9 (56.5–89.7)
Watery stool and (dehydration or rice water stools or vomiting)	98.4 (95.4–99.5)	4.2 (2.6–6.4)	39.6 (36.1–43.1)	80.8 (60.6–93.4)
Watery stool and vomiting and dry mucous membranes	58.7 (53.0–64.2)	55.6 (51.0–60.0)	45.7 (40.8–50.8)	67.8 (63.0–72.4)
Rice water stools	83.1 (78.7–87.0)	12.6 (9.9–15.8)	38.0 (34.5–41.7)	53.7 (44.4–62.8)
Rice water stools and dehydration	84.9 (80.1–88.4)	12.7 (9.9 –16.0)	38.0 (34.4–41.7)	56.6 (47.0–65.9)
Rice water stools and vomiting	76.9 (71.9–81.4)	20.7 (17.2–24.5)	37.9 (34.2–41.8)	58.7 (51.1–66.0)
Rice water stools and ≤ 9 stools in the last 24 hours	39.4 (34.0–45.0)	58.1 (53.6–62.4)	37.5 (32.3–42.9)	60.0 (55.5–64.4)
Rice water stools and (10–25) stools in the last 24 hours	47.3 (41.7–53.0)	53.2 (48.7–57.7)	39.3 (34.3–44.4)	61.3 (56.5–65.9)
Rice water stools and dry mucous membranes	56.5 (50.7–62.0)	52.6 (48.0–57.1)	43.1 (38.2–48.1)	65.5 (60.5–70.2)
Rice water stools and dehydration and vomiting	75.7 (70.6–80.3)	21.0 (17.5–24.8)	37.8 (34.0–41.7)	57.7 (50.2–65.0)
Rice water stools and dehydration and ≤ 9 stools in the last 24 hours	42.0 (36.2–47.9)	56.3 (51.6–60.9)	37.3 (32.0–42.8)	61.0 (56.2–65.7)
Rice water stools and dehydration and (10–25) stools in the last 24 hours	43.3 (38.0–48.7)	55.6 (51.2–60.0)	39.3 (34.3–44.4)	59.7 (55.1–64.1)
Rice water stools and vomiting and dry mucous membranes	50.8 (45.1–56.5)	57.5 (53.0–62.0)	43.3 (38.1–48.5)	64.7 (60.0–69.2)

CI = confidence interval; DRC = Democratic Republic of Congo; NPV = negative predictive value; PPV = positive predictive value.

Supplemental Table 4

Sensitivity, specificity, PPV, and NPV for tested cases aged more than 5 years in Guinea

Case definition	Sensitivity % (95% CI)	Specificity % (95% CI)	PPV % (95% CI)	NPV % (95% CI)
World Health Organization epidemic cholera definition	98.9 (93.6–100.0)	7.8 (4.1–13.2)	37.2 (30.9–43.8)	92.3 (64.0–99.8)
Watery stool and dehydration	86.9 (77.8–93.3)	23.0 (16.5–30.6)	39.0 (32.0–46.4)	75.6 (60.5–87.1)
Watery stool and vomiting	91.7 (83.6–96.6)	15.8 (10.3–22.7)	38.5 (31.7–45.6)	76.7 (57.7–90.1)
Watery stool and ≤ 9 stools in the last 24 hours	87.5 (78.2–93.8)	19.4 (13.0–27.3)	40.2 (32.9–47.9)	71.4 (53.7–85.4)
Watery stool and (10–25) stools in the last 24 hours	11.3 (5.3–20.3)	84.5 (77.1–90.3)	31.0 (15.3–50.8)	60.6 (53.0–67.7)
Watery stool and dry mucous membranes	58.6 (46.2–70.2)	28.6 (21.1–37.0)	30.1 (22.6–38.6)	56.7 (44.0–68.8)
Watery stool and dehydration and vomiting	80.7 (70.6–88.6)	28.5 (21.3–36.6)	39.4 (32.0–47.2)	71.9 (58.5–83.0)
Watery stool and dehydration and ≤ 9 stools in the last 24 hours	46.2 (37.5–55.1)	57.4 (50.0–64.5)	43.0 (34.7–51.5)	60.6 (53.0–67.7)
Watery stool and dehydration and (10–25) stools in the last 24 hours	32.1 (15.9–52.4)	71.2 (58.7–81.7)	32.1 (15.9–52.4)	71.2 (58.7–81.7)
Watery stool and (dehydration or vomiting)	97.6 (91.6–99.7)	10.4 (5.9–16.9)	38.6 (32.0–45.5)	88.2 (63.6–98.5)
Watery stool and (dehydration or rice water stools)	98.8 (93.5–100.0)	15.8 (10.3–22.7)	40.3 (33.5–47.3)	95.8 (78.9–99.9)
Watery stool and (dehydration or rice water stools or vomiting)	98.8 (93.5–100.0)	8.5 (4.4–14.3)	38.7 (32.1–45.6)	92.3 (64.0–99.8)
Watery stool and vomiting and dry mucous membranes	57.1 (44.7–68.9)	34.4 (26.3–43.1)	31.7 (23.7–40.6)	60.0 (48.0–71.1)
Rice water stools	91.8 (83.8–96.6)	49.3 (41.1–57.6)	50.3 (42.2–58.4)	91.5 (83.2–96.5)
Rice water stools and dehydration	79.8 (69.6–87.7)	55.5 (47.0–63.7)	50.8 (41.9–59.6)	82.7 (73.7–89.6)
Rice water stools and vomiting	84.5 (75.0–91.5)	54.2 (45.7–62.5)	51.8 (43.1–60.4)	85.7 (76.8–92.2)
Rice water stools and ≤ 9 stools in the last 24 hours	80.0 (69.6–88.1)	55.1 (46.0–63.9)	52.9 (43.6–62.0)	81.4 (71.6–89.0)
Rice water stools and (10–25) stools in the last 24 hours	11.3 (53–20.3)	89.0 (82.2–93.8)	39.1 (19.7–61.5)	61.4 (54.0–68.5)
Rice water stools and dry mucous membranes	51.4 (39.2–63.6)	61.8 (52.8–70.2)	41.9 (31.3–53.0)	70.4 (31.3–53.0)
Rice water stools and dehydration and vomiting	73.5 (62.7–82.6)	59.2 (50.6–67.3)	51.3 (41.9–60.5)	79.2 (70.3–86.5)
Rice water stools and dehydration and ≤ 9 stools in the last 24 hours	43.7 (34.8–52.8)	71.3 (63.6–78.3)	55.0 (44.7–65.0)	61.2 (53.7–68.3)
Rice water stools and dehydration and (10–25) stools in the last 24 hours	26.5 (12.9–44.4)	85.3 (76.5–91.7)	39.1 (19.7–61.5)	76.4 (67.2–84.1)
Rice water stools and vomiting and dry mucous membranes	50.0 (37.8–62.2)	65.9 (57.0–74.0)	44.3 (33.1–55.9)	70.8 (61.8–78.8)

CI = confidence interval; NPV = negative predictive value; PPV = positive predictive value.

Supplemental Table 5

Sensitivity, specificity, PPV, and NPV for tested cases aged more than 5 years in Mozambique

Case definition	Sensitivity % (95% CI)	Specificity % (95% CI)	PPV % (95% CI)	NPV % (95% CI)
World Health Organization epidemic cholera definition	64.5 (52.7–75.1)	15.6 (11.0–21.1)	21.0 (16.0–26.8)	55.7 (42.4–68.5)
Watery stool and dehydration	66.0 (51.2–78.8)	38.1 (31.9 –45.8)	21.3 (15.1–28.6)	81.9 (72.6–89.1)
Watery stool and vomiting	45.5 (30.4–61.2)	46.7 (39.6–53.9)	16.0 (10.1–23.6)	79.3 (70.8–86.3)
Watery stool and ≤ 9 stools in the last 24 hours	86.7 (69.3–96.2)	17.2 (12.0–23.5)	14.9 (9.9–21.0)	88.6 (73.3–96.8)
Watery stool and (10–25) stools in the last 24 hours	10.0 (2.1–26.5)	91.7 (86.6–95.3)	16.7 (3.6–41.4)	85.9 (80.2–90.5)
Watery stool and dry mucous membranes	11.4 (3.2–26.7)	68.4 (61.0–75.1)	6.7 (1.8–16.2)	79.6 (72.3–85.7)
Watery stool and dehydration and vomiting	38.6 (24.4–54.5)	52.3 (45.0–59.6)	15.6 (9.4–23.8)	78.9 (70.8–85.6)
Watery stool and dehydration and ≤ 9 stools in the last 24 hours	42.6 (28.3–57.8)	63.3 (57.1–69.2)	17.5 (11.1–25.8)	85.7 (79.9–90.4)
Watery stool and dehydration and (10–25) stools in the last 24 hours	23.1 (5.0–53.8)	85.4 (76.7–91.8)	17.6 (3.8–43.4)	89.1 (80.9–94.7)
Watery stool and (dehydration or vomiting)	77.3 (62.2–88.5)	31.6 (25.1–38.7)	20.5 (14.6–27.4)	85.9 (75.6–93.0)
Watery stool and (dehydration or rice water stools)	73.8 (58.0–86.1)	36.8 (30.0–44.1)	20.5 (14.4–27.9)	86.4 (77.0–93.0)
Watery stool and (dehydration or rice water stools or vomiting)	77.5 (61.5–89.2)	30.1 (23.6–37.2)	19.3 (13.5–26.2)	86.2 (75.3–93.5)
Watery stool and vomiting and dry mucous membranes	11.4 (3.2–26.7)	69.9 (62.5–76.6)	7.0 (1.9–17.0)	79.9 (72.7–85.9)
Rice water stools	27.8 (17.9–39.6)	84.2 (78.6–88.8)	37.0 (24.3–51.3)	77.7 (71.8–82.9)
Rice water stools and dehydration	21.4 (10.3–36.8)	85.2 (79.4–89.9)	23.7 (11.4–40.2)	83.5 (77.6–88.4)
Rice water stools and vomiting	20.0 (9.1–35.6)	88.7 (83.4–92.8)	26.7 (12.3–45.9)	84.4 (78.7–89.1)
Rice water stools and ≤ 9 stools in the last 24 hours	38.5 (20.2–59.4)	88.1 (82.3–92.5)	32.3 (16.7–51.4)	90.6 (85.3–94.6)
Rice water stools and (10–25) stools in the last 24 hours	0.0 (0.0–13.2)	94.9 (90.5–97.6)	0.0 (0.0–33.6)	86.5 (80.9–91.0)
Rice water stools and dry mucous membranes	8.8 (1.9–23.7)	93.2 (88.5–96.4)	20.0 (4.3–48.1)	84.2 (78.3–89.0)
Rice water stools and dehydration and vomiting	17.5 (7.3–32.8)	88.5 (83.2–92.7)	24.1 (10.3–43.5)	83.7 (77.9–88.5)
Rice water stools and dehydration and ≤ 9 stools in the last 24 hours	23.5 (10.7–41.2)	90.1 (84.8–94.0)	30.8 (14.3–51.8)	86.3 (80.6–90.9)
Rice water stools and dehydration and (10–25) stools in the last 24 hours	0.0 (0.0–18.5)	95.1 (90.5–97.8)	0.0 (0.0–36.9)	89.5 (84.0–93.7)
Rice water stools and vomiting and dry mucous membranes	8.8 (1.9–23.7)	94.3 (89.8–97.2)	23.1 (5.0–53.8)	84.3 (78.4–89.1)

CI = confidence interval; NPV = negative predictive value; PPV = positive predictive value.

Supplemental Table 6

Sensitivity, specificity, PPV, and NPV for tested cases aged between 1 and 4 years in Mozambique

Case definition	Sensitivity % (95% CI)	Specificity % (95% CI)	PPV % (95% CI)	NPV % (95% CI)
World Health Organization epidemic cholera definition	66.7 (9.4–99.2)	9.4 (3.7–14.7)	1.9 (0.2–6.7)	90.0 (55.5–99.7)
Watery stool and dehydration	100.0 (15.8–100.0)	78.0 (69.0–85.4)	7.7 (0.9–25.1)	100.0 (95.8–100.0)
Watery stool and vomiting	50.0 (1.3–98.7)	57.1 (47.1–66.8)	2.2 (0.1–11.5)	98.4 (91.2–100.0)
Watery stool and ≤ 9 stools in the last 24 hours	0.0 (0.0–97.5)	10.7 (5.5–18.3)	0.0 (0.0–3.9)	91.7 (61.5–99.8)
Watery stool and (10–25) stools in the last 24 hours	100.0 (2.5–100.0)	95.1 (89.0–98.4)	16.7 (0.4–64.1)	100.0 (96.3–100.0)
Watery stool and dry mucous membranes	0.0 (0.0–84.2)	92.1 (85.0–96.5)	0.0 (0.0–36.9)	97.9 (92.6–99.7)
Watery stool and dehydration and vomiting	50.0 (1.3–98.7)	85.6 (77.6–91.7)	6.3 (0.2–30.2)	98.9 (94.0–100.0)
Watery stool and dehydration and ≤ 9 stools in the last 24 hours	NA	NA	NA	NA
Watery stool and dehydration and (10–25) stools in the last 24 hours	50.0 (1.3–98.7)	95.5 (88.8–98.7)	20.0 (0.5–71.6)	98.8 (93.6–100.0)
Watery stool and (dehydration or vomiting)	100.0 (15.8–100.0)	51.0 (41.0–60.9)	3.8 (0.5–13.0)	100.0 (93.3–100.0)
Watery stool and (dehydration or rice water stools)	100.0 (15.8–100.0)	81.7 (72.9–88.6)	9.5 (1.2–30.4)	100.0 (95.8–100.0)
Watery stool and (dehydration or rice water stools or vomiting)	100.0 (15.8–100.0)	53.5 (43.2–63.6)	4.2 (0.5–14.3)	100.0 (93.3–100.0)
Watery stool and vomiting and dry mucous membranes	0.0 (0.0–84.2)	92.9 (85.8–97.1)	0.0 (0.0–41.0)	97.8 (92.4–99.7)
Rice water stools	0.0 (0.0–70.8)	98.1 (93.5–99.8)	0.0 (0.0–84.2)	97.2 (92.2–99.4)
Rice water stools and dehydration	0.0 (0.0–84.2)	98.1 (93.3–99.8)	0.0 (0.0–84.2)	98.1 (93.2–99.8)
Rice water stools and vomiting	0.0 (0.0–84.2)	98.0 (93.0–99.8)	0.0 (0.0–84.2)	98.0 (93.0–99.8)
Rice water stools and ≤ 9 stools in the last 24 hours	0.0 (0.0–97.5)	99.0 (94.6–100.0)	0.0 (0.0–97.5)	99.0 (94.6–100.0)
Rice water stools and (10–25) stools in the last 24 hours	0.0 (0.0–97.5)	99.0 (94.6–100.0)	0.0 (0.0–97.5)	99.0 (94.6–100.0)
Rice water stools and dry mucous membranes	NA	NA	NA	NA
Rice water stools and dehydration and vomiting	0.0 (0.0–84.2)	98.0 (93.0–99.8)	0.0 (0.0–84.2)	98.0 (93.0–99.8)
Rice water stools and dehydration and ≤ 9 stools in the last 24 hours	0.0 (0.0–97.5)	99.0 (94.5–100.0)	0.0 (0.0–97.5)	99.0 (94.5–100.0)
Rice water stools and dehydration and (10–25) stools in the last 24 hours	0.0 (0.0–97.5)	99.0 (94.5–100.0)	0.0 (0.0–97.5)	99.0 (94.5–100.0)
Rice water stools and vomiting and dry mucous membranes	NA	NA	NA	NA

CI = confidence interval; NA = not applicable; NPV = negative predictive value; PPV = positive predictive value.

Supplemental Table 7

Sensitivity, specificity, PPV, and NPV for tested cases aged more than 5 years in Tanzania

Case definition	Sensitivity % (95% CI)	Specificity % (95% CI)	PPV % (95% CI)	NPV % (95% CI)
World Health Organization epidemic cholera definition	91.9 (78.1–98.3)	18.2 (5.2–40.3)	65.4 (50.9–78.0)	57.1 (18.4–90.1)
Watery stool and dehydration	97.1 (84.7–99.9)	25.0 (8.7–49.1)	68.8 (53.7–81.3)	83.3 (35.9–99.6)
Watery stool and vomiting	91.9 (78.1–98.3)	25.0 (8.7–49.1)	69.4 (54.6–81.7)	62.5 (24.5–91.5)
Watery stool and ≤ 9 stools in the last 24 hours	47.1 (29.8–64.9)	31.6 (12.6–56.6)	55.2 (35.7–73.9)	25.0 (9.8–46.7)
Watery stool and (10–25) stools in the last 24 hours	50.0 (32.4–67.6)	84.2 (60.4–96.6)	85.0 (62.1–96.8)	48.5 (30.8–66.5)
Watery stool and dry mucous membranes	33.3 (4.3–77.7)	83.3 (35.9–99.6)	66.7 (9.4–99.2)	55.6 (21.2–86.3)
Watery stool and dehydration and vomiting	97.0 (84.2–99.9)	21.1 (6.1–45.6)	68.8 (53.7–81.3)	80.0 (28.4–99.5)
Watery stool and dehydration and ≤ 9 stools in the last 24 hours	50.0 (31.3–68.7)	57.7 (36.9–76.6)	57.7 (36.9–76.6)	50.0 (31.3–68.7)
Watery stool and dehydration and (10–25) stools in the last 24 hours	50.0 (32.4–67.6)	70.0 (34.8–93.3)	85.0 (62.1–96.8)	29.2 (12.6–51.1)
Watery stool and (dehydration or vomiting)	97.1 (84.7–99.9)	21.1 (6.1–45.6)	68.8 (53.7–81.3)	80.0 (28.4–99.5)
Watery stool and (dehydration or rice water stools)	97.1 (84.7–99.9)	25.0 (8.7–49.1)	68.8 (53.7–81.3)	83.3 (35.9–99.6)
Watery stool and (dehydration or rice water stools or vomiting)	97.1 (84.7–99.9)	21.1 (6.1–45.6)	68.8 (53.7–81.3)	80.0 (28.4–99.5)
Watery stool and vomiting and dry mucous membranes	NA	NA	NA	NA
Rice water stools	83.8 (72.5–96.7)	81.8 (50.1–93.2)	88.6 (72.5–96.7)	75.0 (50.1–93.2)
Rice water stools and dehydration	88.2 (72.5–96.7)	80.0 (56.3–94.3)	88.2 (72.5–96.7)	80.0 (56.3–94.3)
Rice water stools and vomiting	83.8 (68.0–93.8)	80.0 (56.3–94.3)	88.6 (73.3–96.8)	72.7 (49.8–89.3)
Rice water stools and ≤ 9 stools in the last 24 hours	38.2 (22.2–56.4)	94.7 (74.0–99.9)	92.9 (66.1–99.8)	46.2 (30.1–62.8)
Rice water stools and (10–25) stools in the last 24 hours	50.0 (32.4–67.6)	89.5 (66.9–98.7)	89.5 (66.9–98.7)	50.0 (32.4–67.6)
Rice water stools and dry mucous membranes	33.3 (4.3–77.7)	100.0 (54.1–100.0)	100.0 (15.8–100.0)	50.0 (26.2–87.8)
Rice water stools and dehydration and vomiting	88.2 (72.5–96.7)	78.9 (54.4–93.9)	88.2 (72.5–96.7)	78.9 (54.4–93.9)
Rice water stools and dehydration and ≤ 9 stools in the last 24 hours	44.4 (25.5–64.7)	94.1 (71.3–99.9)	92.3 (64.0–99.8)	51.6 (33.1–69.8)
Rice water stools and dehydration and (10–25) stools in the last 24 hours	45.9 (29.5–63.1)	89.5 (66.9–98.7)	89.5 (66.9–98.7)	45.9 (29.5–63.1)
Rice water stools and vomiting and dry mucous membranes	NA	NA	NA	NA

CI = confidence interval; NA = not applicable; NPV = negative predictive value; PPV = positive predictive value.

Supplemental Table 8

Sensitivity, specificity, PPV, and NPV for tested cases aged more than 5 years in Togo

Case definition	Sensitivity % (95% CI)	Specificity % (95% CI)	PPV % (95% CI)	NPV % (95% CI)
World Health Organization epidemic cholera definition	98.9 (96.8–99.8)	6.4 (3.7–10.2)	53.6 (49.1–58.0)	84.2 (60.4–96.6)
Watery stool and dehydration	88.1 (83.7–91.8)	63.5 (57.1–69.6)	72.8 (67.6–77.5)	82.9 (76.7–88.0)
Watery stool and vomiting	91.5 (87.2–94.3)	40.8 (34.6–47.3)	63.1 (58.1–67.9)	80.6 (72.6–87.2)
Watery stool and ≤ 9 stools in the last 24 hours	59.7 (53.4–65.8)	20.4 (15.3–26.2)	45.6 (40.2–51.2)	31.1 (23.7–39.2)
Watery stool and (10–25) stools in the last 24 hours	39.9 (33.8–46.2)	82.7 (77.2–87.4)	72.1 (63.9–79.4)	55.2 (49.7–60.5)
Watery stool and dry mucous membranes	68.5 (62.4–74.3)	76.8 (70.7–82.1)	76.2 (70.1–81.7)	69.2 (63.1–74.8)
Watery stool and dehydration and vomiting	82.5 (77.5–86.9)	69.5 (63.3–75.3)	75.0 (69.7–79.8)	78.2 (72.1–83.6)
Watery stool and dehydration and ≤ 9 stools in the last 24 hours	45.1 (38.2–51.2)	76.8 (71.2–81.8)	67.6 (60.3–74.3)	56.7 (51.3–61.9)
Watery stool and dehydration and (10–25) stools in the last 24 hours	43.9 (37.3–50.7)	86.8 (81.1–91.3)	79.7 (71.5–86.4)	56.7 (50.8–62.5)
Watery stool and (dehydration or vomiting)	97.4 (94.7–98.9)	34.6 (28.6–40.9)	62.2 (57.4–66.9)	92.3 (84.8–96.9)
Watery stool and (dehydration or rice water stools)	95.5 (92.3–97.7)	59.7 (53.1–66.0)	72.7 (67.8–77.3)	92.2 (86.8–95.9)
Watery stool and (dehydration or rice water stools or vomiting)	98.1 (95.7–99.4)	35.0 (29.0–41.5)	63.1 (58.2–67.7)	94.3 (87.2–98.1)
Watery stool and vomiting and dry mucous membranes	65.7 (59.5–71.6)	78.9 (73.1–84.1)	77.3 (71.0–82.7)	67.9 (61.9–73.5)
Rice water stools	70.2 (64.4–75.6)	82.0 (76.6–86.6)	81.3 (75.7–86.1)	71.2 (65.5–76.4)
Rice water stools and dehydration	63.8 (57.7–69.6)	86.1 (81.1–90.3)	83.8 (78.0–88.6)	67.9 (62.3–73.1)
Rice water stools and vomiting	65.1 (59.1–70.7)	84.7 (79.5–89.1)	83.1 (77.4–87.9)	67.8 (62.1–73.1)
Rice water stools and ≤ 9 stools in the last 24 hours	43.3 (37.1–49.6)	85.5 (80.1–89.8)	77.3 (69.5–83.9)	56.8 (51.3–62.2)
Rice water stools and (10–25) stools in the last 24 hours	27.0 (21.6–32.9)	95.5 (91.8–97.8)	87.2 (77.7–93.7)	53.3 (48.2–58.3)
Rice water stools and dry mucous membranes	47.4 (41.0–53.8)	90.2 (85.5–93.7)	84.2 (77.0–89.8)	60.8 (55.4–66.1)
Rice water stools and dehydration and vomiting	59.3 (53.2–65.2)	87.3 (82.4–91.3)	84.1 (78.1–89.0)	65.5 (60.0–70.7)
Rice water stools and dehydration and ≤ 9 stools in the last 24 hours	33.5 (27.9–39.4)	89.7 (85.1–93.3)	79.3 (70.8–86.3)	53.4 (48.4–58.5)
Rice water stools and dehydration and (10–25) stools in the last 24 hours	29.6 (23.7–36.1)	96.0 (92.3–98.3)	89.2 (79.8–95.2)	55.3 (49.9–60.6)
Rice water stools and vomiting and dry mucous membranes	45.3 (39.0–51.8)	91.1 (86.5–94.5)	84.8 (77.6–90.5)	60.2 (54.7–65.4)

CI = confidence interval; NPV = negative predictive value; PPV = positive predictive value.

Supplemental Table 9

Sensitivity, specificity, PPV, and NPV for tested cases aged between 1 and 4 years in Togo

Case definition	Sensitivity % (95% CI)	Specificity % (95% CI)	PPV % (95% CI)	NPV % (95% CI)
World Health Organization epidemic cholera definition	100.0 (81.5–100.0)	10.3 (2.2–27.3)	40.9 (26.3–56.8)	100.0 (29.2–100.0)
Watery stool and dehydration	88.9 (65.3–98.6)	71.4 (51.3–86.8)	66.7 (44.7–84.4)	90.9 (70.8–98.9)
Watery stool and vomiting	94.4 (72.7–99.9)	48.0 (27.8–68.7)	56.7 (37.4–74.5)	92.3 (64.0–99.8)
Watery stool and ≤ 9 stools in the last 24 hours	76.5 (50.1–93.2)	12.0 (2.5–31.2)	37.1 (21.5–55.1)	42.9 (9.9–81.6)
Watery stool and (10–25) stools in the last 24 hours	23.5 (6.8–49.9)	96.0 (79.6–99.9)	80.0 (28.4–99.5)	64.9 (47.5–79.8)
Watery stool and dry mucous membranes	37.5 (15.2–64.6)	84.6 (65.1–95.6)	60.0 (26.2–87.8)	68.8 (50.0–83.9)
Watery stool and dehydration and vomiting	83.3 (58.6–96.4)	84.0 (63.9–95.5)	78.9 (54.4–93.9)	87.5 (67.6–97.3)
Watery stool and dehydration and ≤ 9 stools in the last 24 hours	46.2 (26.6–66.6)	78.1 (60.0–90.7)	63.2 (38.4–83.7)	64.1 (47.2–78.8)
Watery stool and dehydration and (10–25) stools in the last 24 hours	37.5 (8.5–75.5)	100.0 (81.5–100.0)	100.0 (29.2–100.0)	78.3 (56.3–92.5)
Watery stool and (dehydration or vomiting)	100.0 (81.5–100.0)	36.0 (18.0–57.5)	52.9 (35.1–70.2)	100.0 (66.4–100.0)
Watery stool and (dehydration or rice water stools)	100.0 (81.5–100.0)	70.4 (49.8–86.2)	69.2 (48.2–85.7)	100.0 (82.4–100.0)
Watery stool and (dehydration or rice water stools or vomiting)	100.0 (81.5–100.0)	32.0 (14.9–53.5)	51.4 (34.0–68.6)	100.0 (63.1–100.0)
Watery stool and vomiting and dry mucous membranes	37.5 (15.2–64.6)	87.0 (66.4–97.2)	66.7 (29.9–92.5)	66.7 (47.2–82.7)
Rice water stools	72.2 (46.5–90.3)	89.3 (71.8–97.7)	81.3 (54.4–96.0)	83.3 (65.3–94.4)
Rice water stools and dehydration	61.1 (35.7–82.7)	92.6 (75.7–99.1)	84.6 (54.6–98.1)	78.1 (60.0–90.7)
Rice water stools and vomiting	66.7 (41.0–86.7)	96.0 (79.6–99.9)	92.3 (64.0–99.8)	80.0 (61.4–92.3)
Rice water stools and ≤ 9 stools in the last 24 hours	52.9 (27.8–77.0)	87.5 (67.6–97.3)	75.0 (42.8–94.5)	72.4 (52.8–87.3)
Rice water stools and (10–25) stools in the last 24 hours	17.6 (3.8–43.4)	100.0 (85.8–100.0)	100.0 (29.2–100.0)	63.2 (46.0–78.2)
Rice water stools and dry mucous membranes	18.8 (4.0–45.6)	100.0 (86.3– 100.0)	100.0 (29.2–100.0)	65.8 (48.6–80.4)
Rice water stools and dehydration and vomiting	55.6 (30.8–78.5)	96.0 (79.6–99.9)	90.9 (58.7–99.8)	75.0 (56.6–88.5)
Rice water stools and dehydration and ≤ 9 stools in the last 24 hours	34.8 (16.4–57.3)	92.3 (74.9–99.1)	80.0 (44.4–97.5)	61.5 (44.6–76.6)
Rice water stools and dehydration and (10–25) stools in the last 24 hours	18.2 (2.3–51.8)	100.0 (84.6–100.0)	100.0 (15.8–100.0)	71.0 (52.0–85.8)
Rice water stools and vomiting and dry mucous membranes	27.3 (6.0–61.0)	100.0 (84.6–100.0)	100.0 (29.2–100.0)	73.3 (54.1–87.7)

CI = confidence interval; NPV = negative predictive value; PPV = positive predictive value.

Supplemental Table 10

Sensitivity, specificity, PPV and NPV for tested cases aged more than 5 years in Uganda

Case definition	Sensitivity % (95% CI)	Specificity % (95% CI)	PPV % (95% CI)	NPV % (95% CI)
World Health Organization epidemic cholera definition	97.1 (89.8–99.6)	17.6 (12.7–23.5)	27.6 (22.0–33.7)	94.9 (83.7–99.4)
Watery stool and dehydration	91.3 (79.2–97.6)	8.9 (4.5–15.4)	27.3 (20.4–35.0)	73.3 (44.9–92.2)
Watery stool and vomiting	89.1 (76.4–96.4)	23.3 (16.4–31.4)	28.7 (21.4–36.8)	86.1 (70.5–95.3)
Watery stool and ≤ 9 stools in the last 24 hours	33.3 (17.3–52.8)	42.2 (29.9–55.2)	21.3 (10.7–35.7)	57.4 (42.2–71.7)
Watery stool and (10–25) stools in the last 24 hours	66.7 (47.2–82.7)	64.1 (51.1–75.7)	46.5 (31.2–62.3)	80.4 (66.9–90.2)
Watery stool and dry mucous membranes	66.7 (9.4–99.2)	41.0 (25.6–57.9)	8.0 (1.0–26.0)	94.1 (71.3–99.9)
Watery stool and dehydration and vomiting	83.8 (68.0–93.8)	20.0 (12.7–29.2)	27.9 (19.8–37.2)	76.9 (56.4–91.0)
Watery stool and dehydration and ≤ 9 stools in the last 24 hours	45.5 (16.7–76.6)	55.2 (41.5–68.3)	16.1 (5.5–33.7)	84.2 (68.7–94.0)
Watery stool and dehydration and (10–25) stools in the last 24 hours	48.4 (30.2–66.9)	57.1 (41.0–72.3)	45.5 (28.1–63.6)	60.0 (43.3–75.1)
Watery stool and (dehydration or vomiting)	94.6 (81.8–99.3)	10.0 (4.9–17.6	28.0 (20.3–36.7)	83.3 (51.6–97.9)
Watery stool and (dehydration or rice water stools)	97.6 (87.1–99.9)	8.3 (3.8–15.1)	28.6 (21.3–36.8)	90.0 (55.5–99.7)
Watery stool and (dehydration or rice water stools or vomiting)	96.9 (83.8–99.9)	9.1 (4.0–17.1)	27.9 (19.8–37.2)	88.9 (51.8–99.7)
Watery stool and vomiting and dry mucous membranes	50.0 (1.3–98.7)	48.7 (32.4–65.2)	4.8 (0.1–23.8)	95.0 (75.1–99.9)
Rice water stools	73.3 (60.3–83.9)	49.4 (41.8–57.1)	33.3 (25.4–42.1)	84.3 (75.8–90.8)
Rice water stools and dehydration	71.4 (55.4–84.3)	40.7 (31.6–50.4)	30.9 (21.9–41.1)	79.3 (66.6–88.8)
Rice water stools and vomiting	70.7 (54.5–83.9)	45.4 (35.8–55.2)	33.0 (23.3–43.8)	80.3 (68.2–89.4)
Rice water stools and ≤ 9 stools in the last 24 hours	27.6 (12.7–47.2)	71.7 (58.6–82.5)	32.0 (14.9–53.5)	67.2 (54.3–78.4)
Rice water stools and (10–25) stools in the last 24 hours	51.7 (32.5–70.6)	70.0 (56.8–81.2)	45.5 (28.1–63.6)	75.0 (61.6–85.6)
Rice water stools and dry mucous membranes	66.7 (9.4–99.2)	58.3 (40.8–74.5)	11.8 (1.5–36.4)	95.5 (77.2–99.9)
Rice water stools and dehydration and vomiting	69.7 (51.3–84.4)	45.7 (35.2–56.4)	31.5 (21.1–43.4)	80.8 (67.5–90.4)
Rice water stools and dehydration and ≤ 9 stools in the last 24 hours	35.7 (12.8–64.9)	72.7 (57.2–85.0)	29.4 (10.3–56.0)	78.0 (62.4–89.4)
Rice water stools and dehydration and (10–25) stools in the last 24 hours	42.3 (23.4–63.1)	70.0 (55.4–82.1)	42.3 (23.4–63.1)	70.0 (55.4–82.1)
Rice water stools and vomiting and dry mucous membranes	50.0 (1.3–98.7)	61.1 (43.5–76.9)	6.7 (0.2–31.9)	95.7 (78.1–99.9)

CI = confidence interval; NPV = negative predictive value; PPV = positive predictive value.

Supplemental Table 11

Sensitivity, specificity, PPV, and NPV for tested cases aged between 1 and 4 years in Uganda

Case definition	Sensitivity % (95% CI)	Specificity % (95% CI)	PPV % (95% CI)	NPV % (95% CI)
World Health Organization epidemic cholera definition	92.9 (66.1–99.8)	13.0 (2.8–33.6)	39.4 (22.9–57.9)	75.0 (19.4–99.4)
Watery stool and dehydration	90.9 (58.7–99.8)	0.0 (0.0–20.6)	38.5 (20.2–59.4)	0.0 (0.0–97.5)
Watery stool and vomiting	91.7 (61.5–99.8)	12.5 (1.6–38.3)	44.0 (24.4–65.1)	66.7 (9.4–99.2)
Watery stool and ≤ 9 stools in the last 24 hours	60.0 (26.2–87.8)	33.3 (4.3–77.7)	60.0 (26.2–87.8)	33.3 (4.3–77.7)
Watery stool and (10–25) stools in the last 24 hours	30.0 (6.7–65.2)	66.7 (22.3–95.7)	60.0 (14.7–94.7)	36.4 (10.9–69.2)
Watery stool and dry mucous membranes	NA	NA	NA	NA
Watery stool and dehydration and vomiting	88.9 (51.8–99.7)	14.3 (1.8–42.8)	40.0 (19.1–63.9)	66.7 (9.4–99.2)
Watery stool and dehydration and ≤ 9 stools in the last 24 hours	44.4 (13.7–78.8)	50.0 (11.8–88.2)	57.1 (18.4–90.1)	37.5 (8.5–75.5)
Watery stool and dehydration and (10–25) stools in the last 24 hours	42.9 (9.9–81.6)	50.0 (6.8–93.2)	60.0 (14.7–94.7)	33.3 (4.3–77.7)
Watery stool and (dehydration or vomiting)	88.9 (51.8–99.7)	0.0 (0.0–23.2)	36.4 (17.2–59.3)	0.0 (0.0–97.5)
Watery stool and (dehydration or rice water stools)	88.9 (51.8–99.7)	0.0 (0.0–24.7)	38.1 (20.3–66.5)	0.0 (0.0–97.5)
Watery stool and (dehydration or rice water stools or vomiting)	85.7 (42.1–99.6)	0.0 (0.0–30.8)	37.5 (18.1–61.6)	0.0 (0.0–97.5)
Watery stool and vomiting and dry mucous membranes	NA	NA	NA	NA
Rice water stools	54.5 (23.4–83.3)	42.1 (20.3–66.5)	35.3 (14.2–61.7)	61.5 (14.2–86.1)
Rice water stools and dehydration	44.5 (13.7–78.8)	33.3 (11.8–61.6)	28.6 (8.4–58.1)	50.0 (18.7–81.3)
Rice water stools and vomiting	44.4 (13.7–78.8)	53.8 (25.1–80.8)	40.0 (12.2–73.8)	58.3 (27.7–84.8)
Rice water stools and ≤ 9 stools in the last 24 hours	33.3 (7.5–70.1)	50.0 (11.8–88.2)	50.0 (11.8–88.2)	33.3 (7.5–70.1)
Rice water stools and (10–25) stools in the last 24 hours	33.3 (7.5–70.1)	66.7 (22.3–95.7)	60.0 (14.7–94.7)	40.0 (12.2–73.8)
Rice water stools and dry mucous membranes	NA	NA	NA	NA
Rice water stools and dehydration and vomiting	28.6 (3.7–71.0)	58.3 (27.7–84.8)	28.6 (3.7–71.0)	58.3 (27.7–84.8)
Rice water stools and dehydration and ≤ 9 stools in the last 24 hours	20.0 (0.5–71.6)	60.0 (14.7–94.7)	33.3 (0.8–90.6)	42.9 (9.9–81.6)
Rice water stools and dehydration and (10–25) stools in the last 24 hours	33.3 (7.5–70.1)	60.0 (14.7–94.7)	60.0 (14.7–94.7)	33.3 (7.5–70.1)
Rice water stools and vomiting and dry mucous membranes	NA	NA	NA	NA

CI = confidence interval; NA = not applicable; NPV = negative predictive value; PPV = positive predictive value.

Supplemental Table 12

Sensitivity, specificity, PPV, and NPV for tested cases aged more than 5 years in the enhanced surveillance zones

Case definition	Sensitivity % (95% CI)	Specificity % (95% CI)	PPV % (95% CI)	NPV % (95% CI)
World Health Organization epidemic cholera definition	97.8 (96.9–98.6)	6.5 (5.4–7.6)	35.3 (33.6–37.0)	85.2 (78.6–90.4)
Watery stool and dehydration	93.0 (91.3–94.5)	15.9 (14.3–17.6)	37.4 (35.5–39.3)	80.8 (76.5–84.7)
Watery stool and vomiting	90.4 (88.5–92.1)	20.6 (18.9–22.5)	37.9 (36.0–39.8)	80.1 (76.3–83.5)
Watery stool and ≤ 9 stools in the last 24 hours	47.7 (44.5–50.8)	50.9 (48.6–53.2)	34.5 (32.0–37.1)	64.2 (61.6–66.6)
Watery stool and (10–25) stools in the last 24 hours	50.7 (47.5–53.9)	52.8 (50.5–55.1)	36.9 (34.3–39.5)	66.3 (63.9–68.8)
Watery stool and dry mucous membranes	74.6 (71.7–77.3)	31.1 (28.9–33.3)	37.0 (34.9–39.2)	69.2 (65.9–72.4)
Watery stool and dehydration and vomiting	86.5 (84.3–88.5)	25.1 (23.2–27.1)	38.6 (36.6–40.6)	77.5 (74.0–80.7)
Watery stool and dehydration and ≤ 9 stools in the last 24 hours	46.9 (43.6–50.1)	57.8 (55.4–60.2)	38.2 (35.3–41.1)	66.2 (63.7–68.6)
Watery stool and dehydration and (10–25) stools in the last 24 hours	47.2 (44.2–50.3)	56.7 (54.4–58.9)	37.1 (34.5–39.7)	66.5 (64.2–68.8)
Watery stool and (dehydration or vomiting)	97.5 (96.4–98.4)	10.4 (9.1–11.9)	37.1 (35.3–39.0)	88.4 (83.5–92.3)
Watery stool and (dehydration or rice water stools)	96.6 (95.3–97.6)	14.3 (12.8–16.0)	38.1 (36.2–40.0)	88.6 (84.5–91.9)
Watery stool and (dehydration or rice water stools or vomiting)	98.0 (96.9–98.7)	10.2 (8.8–11.6)	37.5 (35.6–39.3)	90.1 (85.3–93.8)
Watery stool and vomiting and dry mucous membranes	70.1 (67.1–73.0)	38.0 (35.7–40.3)	38.2 (35.9–40.5)	70.0 (67.0–72.9)
Rice water stools	80.5 (77.9–82.8)	34.6 (32.5–36.7)	39.5 (37.4–41.6)	76.9 (74.0–79.6)
Rice water stools and dehydration	77.6 (74.9–80.1)	33.8 (31.7–36.0)	39.0 (36.9–41.1)	73.5 (70.4–76.4)
Rice water stools and vomiting	75.1 (72.4–77.7)	40.0 (37.8–42.2)	40.6 (38.4–42.8)	74.6 (71.9–77.3)
Rice water stools and ≤ 9 stools in the last 24 hours	38.0 (35.0–41.1)	72.4 (70.3–74.5)	43.0 (39.7–49.4)	68.1 (65.9–70.1)
Rice water stools and (10–25) stools in the last 24 hours	43.5 (40.4–46.7)	58.8 (56.5–61.1)	36.7 (33.9–39.5)	65.5 (63.1–67.8)
Rice water stools and dry mucous membranes	62.7 (59.6–69.8)	43.4 (41.1–45.8)	37.8 (35.4–40.2)	68.0 (65.2–70.7)
Rice water stools and dehydration and vomiting	72.5 (69.7–75.2)	40.4 (38.2–42.7)	40.0 (37.8–42.3)	72.8 (70.0–75.5)
Rice water stools and dehydration and ≤ 9 stools in the last 24 hours	38.4 (35.2–41.7)	68.6 (66.2–70.9)	42.2 (38.8–45.6)	65.1 (62.7–67.4)
Rice water stools and dehydration and (10–25) stools in the last 24 hours	40.1 (37.1–43.1)	63.8 (61.7–65.9)	36.5 (33.7–39.3)	67.2 (65.1–69.3)
Rice water stools and vomiting and dry mucous membranes	59.9 (56.8–63.0)	48.6 (46.3–51.0)	39.0 (36.5–41.5)	68.9 (66.2–71.4)

CI = confidence interval; NPV = negative predictive value; PPV = positive predictive value.

Supplemental Table 13

Sensitivity, specificity, PPV, and NPV for tested cases aged between 1 and 4 years in the enhanced surveillance zones

Case definition	Sensitivity % (95% CI)	Specificity % (95% CI)	PPV % (95% CI)	NPV % (95% CI)
World Health Organization epidemic cholera definition	97.7 (95.5–99.0)	5.3 (3.7–7.3)	35.7 (32.6–38.8)	81.0 (65.9–91.4)
Watery stool and dehydration	95.6 (92.8–97.5)	18.3 (15.4–21.6)	38.9 (35.6–42.3)	88.5 (81.7–93.4)
Watery stool and vomiting	87.8 (83.9–91.1)	23.4 (20.1–26.9)	38.7 (35.2–42.2)	77.7 (71.0–83.4)
Watery stool and ≤ 9 stools in the last 24 hours	49.6 (44.1–55.0)	45.3 (41.3–49.3)	33.3 (29.2–37.6)	62.0 (57.3–66.5)
Watery stool and (10–25) stools in the last 24 hours	49.0 (43.5–54.4)	58.5 (54.5–62.4)	39.4 (34.7–44.2)	67.5 (63.4–71.5)
Watery stool and dry mucous membranes	64.3 (58.9–69.5)	49.0 (44.5–53.5)	45.3 (40.7–49.9)	67.7 (62.6–72.5)
Watery stool and dehydration and vomiting	85.9 (81.7–89.4)	28.2 (24.7–32.0)	39.8 (36.2–43.2)	78.4 (72.4–83.6)
Watery stool and dehydration and ≤ 9 stools in the last 24 hours	48.2 (42.7–53.7)	58.6 (54.6–62.6)	38.9 (34.2–43.8)	67.4 (63.2–71.4)
Watery stool and dehydration and (10–25) stools in the last 24 hours	48.2 (42.0–53.7)	58.7 (54.7–62.7)	39.4 (34.7–44.3)	67.0 (62.9–71.0)
Watery stool and (dehydration or vomiting)	97.9 (95.8–99.2)	12.9 (10.4–15.8)	38.0 (34.8–41.3)	92.0 (84.3–96.7)
Watery stool and (dehydration or rice water stools)	97.6 (95.4–99.0)	19.6 (16.6–22.9)	39.6 (36.3–43.0)	93.9 (88.3–97.3)
Watery stool and (dehydration or rice water stools or vomiting)	98.2 (96.2–99.3)	13.0 (10.4–15.9)	38.2 (35.0–41.5)	93.0 (85.4–97.4)
Watery stool and vomiting and dry mucous membranes	57.8 (52.2–63.2)	61.9 (57.8–65.8)	45.5 (40.7–50.5)	72.7 (68.6–76.5)
Rice water stools	83.3 (79.0–87.1)	28.9 (25.4–32.6)	38.8 (35.2–42.4)	76.3 (70.4–81.5)
Rice water stools and dehydration	81.7 (77.2–85.7)	28.4 (24.9–32.1)	38.4 (34.9–42.1)	73.9 (67.9–79.4)
Rice water stools and vomiting	74.5 (69.5–79.0)	35.1 (31.3–39.0)	38.8 (35.0–42.6)	71.4 (65.9–76.4)
Rice water stools and ≤ 9 stools in the last 24 hours	38.9 (33.7–44.4)	65.7 (61.7–69.5)	38.5 (33.2–43.9)	66.1 (62.2–69.9)
Rice water stools and (10–25) stools in the last 24 hours	45.5 (40.1–50)	61.7 (57.7–65.6)	39.6 (34.7–44.7)	67.3 (63.2–71.2)
Rice water stools and dry mucous membranes	53.8 (48.3–59.3)	53.0 (48.5–57.5)	43.1 (38.3–48.1)	63.4 (58.6–58.1)
Rice water stools and dehydration and vomiting	73.1 (68.0–77.7)	34.8 (31.0–38.7)	38.3 (34.5–42.2)	70.0 (64.5–75.1)
Rice water stools and dehydration and ≤ 9 stools in the last 24 hours	40.7 (35.2–46.2)	64.2 (60.2–68.6)	37.8 (32.5–43.2)	67.0 (62.9–70.9)
Rice water stools and dehydration and (10–25) stools in the last 24 hours	42.0 (36.8–47.3)	63.2 (59.3–67.0)	39.4 (34.5–44.5)	65.6 (61.7–69.4)
Rice water stools and vomiting and dry mucous membranes	48.5 (42.9–54.0)	64.9 (60.8–68.7)	43.3 (38.1–48.5)	69.5 (65.4–73.3)

CI = confidence interval; NPV = negative predictive value; PPV = positive predictive value.

Supplemental Table 14

Sensitivity, specificity, PPV, and NPV for tested cases aged more than 5 years in outbreak sites

Case definition	Sensitivity % (95% CI)	Specificity % (95% CI)	PPV % (95% CI)	NPV % (95% CI)
World Health Organization epidemic cholera definition	75.8 (70.7–80.4)	15.8 (12.5–19.6)	39.9 (36.0–43.9)	46.9 (38.7–55.3)
Watery stool and dehydration	87.9 (82.8–91.9)	25.8 (21.1–30.8)	43.5 (38.8–48.4)	76.6 (67.6–84.1)
Watery stool and vomiting	81.1 (75.3–86.0)	22.7 (18.4–27.5)	40.4 (35.9–45.2)	65.0 (55.8–73.5)
Watery stool and ≤ 9 stools in the last 24 hours	69.7 (62.6–76.2)	23.8 (18.9–29.2)	37.9 (32.7–43.2)	54.0 (44.9–63.0)
Watery stool and (10–25) stools in the last 24 hours	27.7 (21.4–34.6)	80.1 (75.0–84.6)	48.1 (38.4–58.0)	62.4 (57.2–67.4)
Watery stool and dry mucous membranes	53.6 (44.5–62.6)	56.7 (50.2–63.1)	39.4 (32.0–47.2)	69.9 (62.9–76.3)
Watery stool and dehydration and vomiting	76.6 (70.2–82.2)	35.6 (30.3–41.2)	43.9 (38.6–49.2)	69.8 (62.0–76.8)
Watery stool and dehydration and ≤ 9 stools in the last 24 hours	46.4 (39.9–53.0)	60.2 (54.9–65.3)	43.9 (37.6–50.2)	62.6 (57.3–67.8)
Watery stool and dehydration and (10–25) stools in the last 24 hours	41.9 (32.3–51.9)	73.1 (65.4–79.9)	51.2 (40.1–62.1)	65.1 (57.6–72.2)
Watery stool and (dehydration or vomiting)	92.7 (88.2–95.8)	12.5 (9.0–16.7)	41.0 (36.5–45.7)	72.2 (58.4–83.5)
Watery stool and (dehydration or rice water stools)	92.4 (88.0–95.6)	16.0 (12.1–20.4)	41.6 (37.1–46.2)	76.5 (64.6–85.9)
Watery stool and (dehydration or rice water stools or vomiting)	93.0 (88.6–96.1)	9.1 (6.1–12.9)	40.0 (35.6–44.6)	66.7 (50.5–80.4)
Watery stool and vomiting and dry mucous membranes	50.8 (41.7–59.9)	61.7 (55.2–67.9)	41.2 (33.3–49.4)	70.4 (63.6–76.5)
Rice water stools	55.8 (50.2–61.4)	46.2 (41.4–51.0)	43.3 (38.4–48.2)	58.7 (53.3–64.0)
Rice water stools and dehydration	65.9 (59.1–72.2)	49.4 (43.9–54.9)	45.3 (39.6–51.0)	45.3 (63.2–75.3)
Rice water stools and vomiting	65.1 (58.4–71.4)	48.2 (42.8–53.7)	44.5 (39.0–50.2)	68.5 (62.2–74.3)
Rice water stools and ≤ 9 stools in the last 24 hours	52.7 (45.2–60.1)	51.6 (45.6–57.6)	41.8 (35.4–48.4)	62.3 (55.7–68.6)
Rice water stools and (10–25) stools in the last 24 hours	22.8 (17.0–29.6)	84.2 (79.4–88.3)	48.8 (37.9–59.9)	62.3 (57.2–67.2)
Rice water stools and dry mucous membranes	46.8 (37.8–55.9)	65.5 (59.1–71.6)	41.4 (33.2–50.1)	70.3 (63.8–76.2)
Rice water stools and dehydration and vomiting	62.2 (55.1–68.9)	54.8 (49.1–60.4)	46.8 (40.7–53.0)	69.4 (63.2–75.0)
Rice water stools and dehydration and ≤ 9 stools in the last 24 hours	39.2 (32.6–46.0)	69.7 (64.3–74.8)	47.2 (39.8–54.8)	62.4 (57.1–67.5)
Rice water stools and dehydration and (10–25) stools in the last 24 hours	29.9 (21.8–39.1)	82.6 (76.5–87.7)	51.5 (39.0–63.8)	65.7 (59.3–71.7)
Rice water stools and vomiting and dry mucous membranes	43.9 (35.0–53.1)	69.8 (63.5–75.6)	43.2 (34.4–52.4)	70.4 (64.1–76.2)

CI = confidence interval; NPV = negative predictive value; PPV = positive predictive value.

Supplemental Table 15

Sensitivity, specificity, PPV, and NPV for tested cases aged between 1 and 4 years in outbreak sites

Case definition	Sensitivity % (95% CI)	Specificity % (95% CI)	PPV % (95% CI)	NPV % (95% CI)
World Health Organization epidemic cholera definition	62.9 (44.9–78.5)	23.1 (11.1–39.3)	42.3 (28.7–56.8)	40.9 (20.7–63.6)
Watery stool and dehydration	85.0 (62.1–96.8)	34.5 (17.9–54.3)	47.2 (30.4–64.5)	76.9 (46.2–95.0)
Watery stool and vomiting	90.5 (69.6–98.8)	20.0 (7.7–38.6)	44.2 (29.1–60.1)	75.0 (34.9–96.8)
Watery stool and ≤ 9 stools in the last 24 hours	66.7 (41.0–86.7)	22.7 (7.8–45.4)	41.4 (23.5–61.1)	45.5 (16.7–76.6)
Watery stool and (10–25) stools in the last 24 hours	27.8 (9.7–53.5)	86.4 (65.1–97.1)	62.5 (24.5–91.5)	59.4 (40.6–76.3)
Watery stool and dry mucous membranes	45.5 (16.7–76.6)	55.6 (30.8–78.5)	38.5 (13.9–68.4)	62.5 (35.4–84.8)
Watery stool and dehydration and vomiting	77.8 (52.4–93.6)	39.3 (21.5–59.4)	45.2 (27.3–64.0)	73.3 (44.9–92.2)
Watery stool and dehydration and ≤ 9 stools in the last 24 hours	42.1 (20.3–66.5)	65.4 (44.3–82.8)	47.1 (23.0–72.2)	60.7 (40.6–78.5)
Watery stool and dehydration and (10–25) stools in the last 24 hours	38.5 (13.9–68.4)	78.6 (49.2–95.3)	62.5 (24.5–91.5)	57.9 (33.5–79.7)
Watery stool and (dehydration or vomiting)	94.4 (72.7–99.9)	14.3 (4.0–32.7)	41.5 (26.3–57.9)	80.0 (28.4–99.5)
Watery stool and (dehydration or rice water stools)	89.5 (66.9–98.7)	17.2 (5.8–35.8)	41.5 (26.3–57.9)	71.4 (29.0–96.3)
Watery stool and (dehydration or rice water stools or vomiting)	94.1 (71.3–99.9)	10.7 (2.3–28.2)	39.0 (24.2–55.5)	75.0 (19.4–99.4)
Watery stool and vomiting and dry mucous membranes	45.5 (16.7–76.6)	52.9 (27.8–77.0)	38.5 (13.9–68.4)	60.0 (32.3–83.7)
Rice water stools	38.2 (22.2–56.4)	53.7 (37.4–69.3)	40.6 (23.7–59.4)	51.2 (35.5–66.7)
Rice water stools and dehydration	52.6 (28.9–75.6)	61.3 (42.2–78.2)	45.5 (24.4–67.8)	67.9 (47.6–84.1)
Rice water stools and vomiting	55.0 (31.5–76.9)	51.6 (33.1–68.9)	42.3 (23.4–63.1)	64.0 (42.5–82.0)
Rice water stools and ≤ 9 stools in the last 24 hours	50.0 (26.0–74.0)	50.0 (28.2–71.8)	45.0 (23.1–68.5)	55.0 (31.5–76.9)
Rice water stools and (10–25) stools in the last 24 hours	22.2 (6.4–47.6)	90.9 (70.8–98.9)	66.7 (22.3–95.7)	58.8 (40.7–75.4)
Rice water stools and dry mucous membranes	36.4 (10.9–69.2)	68.4 (43.4–87.4)	40.0 (12.2–73.8)	65.0 (40.8–84.6)
Rice water stools and dehydration and vomiting	47.1 (23.0–72.2)	69.0 (49.2–84.7)	47.1 (23.0–72.2)	69.0 (49.2–84.7)
Rice water stools and dehydration and ≤ 9 stools in the last 24 hours	33.3 (13.3–59.0)	78.3 (56.3–92.5)	54.5 (23.4–83.3)	60.0 (40.6–77.3)
Rice water stools and dehydration and (10–25) stools in the last 24 hours	28.6 (8.4–58.1)	88.2 (63.6–98.5)	66.7 (22.3–95.7)	60.0 (38.7–78.9)
Rice water stools and vomiting and dry mucous membranes	36.4 (10.9–69.2)	66.7 (41.0–86.7)	40.0 (12.2–73.8)	63.2 (38.4–83.7)

CI = confidence interval; NPV = negative predictive value; PPV = positive predictive value.

Supplemental Table 16

Sensitivity, specificity, PPV, and NPV for the World Health Organization case definition; children aged 1–4, 1–3, and 1–2

Case definition	Sensitivity % (95% CI)	Specificity % (95% CI)	PPV % (95% CI)	NPV % (95% CI)
Less than 5	92.7 (91.2–94.0)	8.1 (7.1–9.3)	36.1 (34.5–37.7)	66.6 (60.9–71.9)
Less than 4	94.4 (91.1–96.8)	6.6 (4.7–9.0)	34.3 (31.0–37.1)	69.8 (55.7–81.7)
Less than 3	93.4 (88.5–96.7)	6.7 (4.4–9.5)	29.2 (25.3–33.2)	71.1 (54.1–84.6)
Less than 2	90.9 (80.0–97.0)	8.6 (5.0–13.7)	22.8 (17.4–29.0)	76.2 (52.8–91.8)

CI = confidence interval; NPV = negative predictive value; PPV = positive predictive value.

Supplemental Table 17

Sensitivity, specificity, PPV, and NPV of country specific case definitions

Case definition	Sensitivity % (95% CI)	Specificity % (95% CI)	PPV % (95% CI)	NPV % (95% CI)
Côte d'Ivoire	46.5 (31.2–62.3)	56.8 (45.3–67.8)	36.4 (23.8–50.4)	66.7 (54.3–77.6)
DRC	94.8 (93.3–96.0)	7.7 (6.6–9.0)	35.7 (33.9–37.4)	73.5 (66.8–79.1)
Guinea	95.7 (89.5–98.8)	8.6 (4.8–14.0)	37.7 (31.5–44.1)	77.8 (52.4–93.6)
Mozambique	100.0 (91.8–100.0)	4.1 (2.0–7.5)	15.7 (11.6–20.6)	100.0 (69.2–100.0)
Tanzania	92.3 (79.1–98.4)	16.7 (4.7–37.4)	64.3 (50.4–76.6)	57.1 (18.4–90.1)
Togo	87.5 (83.0–91.2)	63.0 (56.6–69.1)	72.4 (67.3–77.2)	82.0 (75.8–87.2)
Uganda	91.3 (82.8–96.4)	4.1 (1.8–8.2)	28.2 (22.8–34.1)	53.3 (26.6–78.7)

CI = confidence interval; DRC = Democratic Republic of Congo; NPV = negative predictive value; PPV = positive predictive value.
